# Endophilin A2 deficiency protects rodents from autoimmune arthritis by modulating T cell activation

**DOI:** 10.1038/s41467-020-20586-2

**Published:** 2021-01-27

**Authors:** Ulrika Norin, Carola Rintisch, Liesu Meng, Florian Forster, Diana Ekman, Jonatan Tuncel, Katrin Klocke, Johan Bäcklund, Min Yang, Michael Y. Bonner, Gonzalo Fernandez Lahore, Jaime James, Klementy Shchetynsky, Maria Bergquist, Inger Gjertsson, Norbert Hubner, Liselotte Bäckdahl, Rikard Holmdahl

**Affiliations:** 1 Medical Inflammation Research, Department of Medical Biochemistry and Biophysics, Karolinska Institute, Stockholm, Sweden; 2 Medical Inflammation Research, Lund University, Lund, Sweden; 3 Cardiovascular and Metabolic Sciences, Max-Delbrück-Center for Molecular Medicine (MDC), Berlin, Germany; 4 The Second affiliated hospital to Xi’an Jiaotong University and the Department of Biochemistry and Molecular Biology, School of Basic Medical Sciences, Xi’an Jiaotong University Health Science Center, 710061 Xi’an, Shaanxi China; 5 Department of Biochemistry and Biophysics, National Bioinformatics Infrastructure Sweden, Science for Life Laboratory, Stockholm University, Stockholm, Sweden; 6 Rheumatology Unit, Department of Medicine, Karolinska Institute and Karolinska University Hospital, Stockholm, Sweden; 7 Department of Rheumatology and Inflammation Research, Institute for Medicine, Sahlgrenska Academy, University of Gothenburg, Gothenburg, Sweden; 8 Department of Medical Sciences, Clinical Physiology, Uppsala University, Uppsala, Sweden; 9 Charité-Universitätsmedizin Berlin, Berlin, Germany

**Keywords:** Adaptive immunity, Lymphocyte activation, Autoimmunity, T cells

## Abstract

The introduction of the CTLA-4 recombinant fusion protein has demonstrated therapeutic effects by selectively modulating T-cell activation in rheumatoid arthritis. Here we show, using a forward genetic approach, that a mutation in the *SH3gl1* gene encoding the endocytic protein Endophilin A2 is associated with the development of arthritis in rodents. Defective expression of *SH3gl1* affects T cell effector functions and alters the activation threshold of autoreactive T cells, thereby leading to complete protection from chronic autoimmune inflammatory disease in both mice and rats. We further show that *SH3GL1* regulates human T cell signaling and T cell receptor internalization, and its expression is upregulated in rheumatoid arthritis patients. Collectively our data identify SH3GL1 as a key regulator of T cell activation, and as a potential target for treatment of autoimmune diseases.

## Abstract (web summary)

The autoimmune disorder, rheumatoid arthritis (RA), has been associated with multiple pathophysiological factors. Here the authors show that deficiency in endophilin A2 in rodents protects them from experimental arthritis by altering T cell activation threshold and effector functions, thereby hinting a potential target for RA therapy.

## Introduction

Aberrant activation of autoreactive T cells has been suggested to initiate and drive autoimmune diseases, such as rheumatoid arthritis (RA) and multiple sclerosis (MS). It is believed that RA is initiated years before the clinical onset through an activation of major histocompatibility complex (MHC) class II-restricted autoreactive T cells that give specific help to B cells, leading to production of autoantibodies^1^. In RA, the chronic inflammatory attack of the joints, initiated by the adaptive immune system, is followed by an increased production of inflammatory cytokines such as TNF and IL-6 and during the last decades effective pharmaceutical treatments neutralizing this late inflammatory phase have been developed. Although early treatment has been shown to be more effective, as it improves the possibility to interfere with the underlying cause of autoimmune diseases, patients receive treatment well after disease is established^2^. Thus, a better understanding of the role of the adaptive immune system that drives the early stages of the disease i.e. before the disease is clinically overt, is needed to improve treatment of autoimmune diseases.

Genetic predisposition of RA is known to be strongly associated with MHC class II genes implicating a major contribution of T cells. Additionally, a recent genome wide meta-analysis revealed over 100 non-MHC risk loci where many were suggested to be related to T cell immune functions^3^. T cells can be found in abundance in the synovium of RA patients^4^ and the successful use of T cell targeting therapy, such as the CTLA-4-Ig fusion antibody abatacept, confirm the importance of T cells in the chronic stage of the disease. These observations are corroborated in animal models for RA, where depletion of T cells by use of antibodies or gene deletion protects against disease induction^5,6^. A specific role of the T cell receptor (TCR) in regulating development of arthritis has been further illustrated in the SKG mouse^7^, where a mutation in TCR signaling molecule Zap70 leads to altered TCR signaling. This in turn skews T cell development in the thymus increasing the number of autoreactive T cells leading to subsequent development of arthritis.

Herein, we have identified Endophilin A2 (EA2), encoded by the *SH3gl1* gene, as a regulator of TCR internalization, signaling and downstream T cell effector functions. So far, EA2 has predominantly been studied in synaptic transmission in the central nervous system and in cancer^8–10^. We demonstrate that deficient expression of *SH3gl1*, caused by either a spontaneous mutation in the DA rat, or in genetically modified mice, leads to protection against autoimmunity. We hereby report that EA2 has a fundamental role in autoimmunity and limits the induction of autoreactive T cells. The discovery of the EA2’s impact on T cell activation opens up to explore new pathways and treatment possibilities for not only RA but for all T cell dependent inflammatory diseases.

## Results

### A spontaneous mutation in the SH3g11 gene protects against autoimmune arthritis

**Fig. 1:**
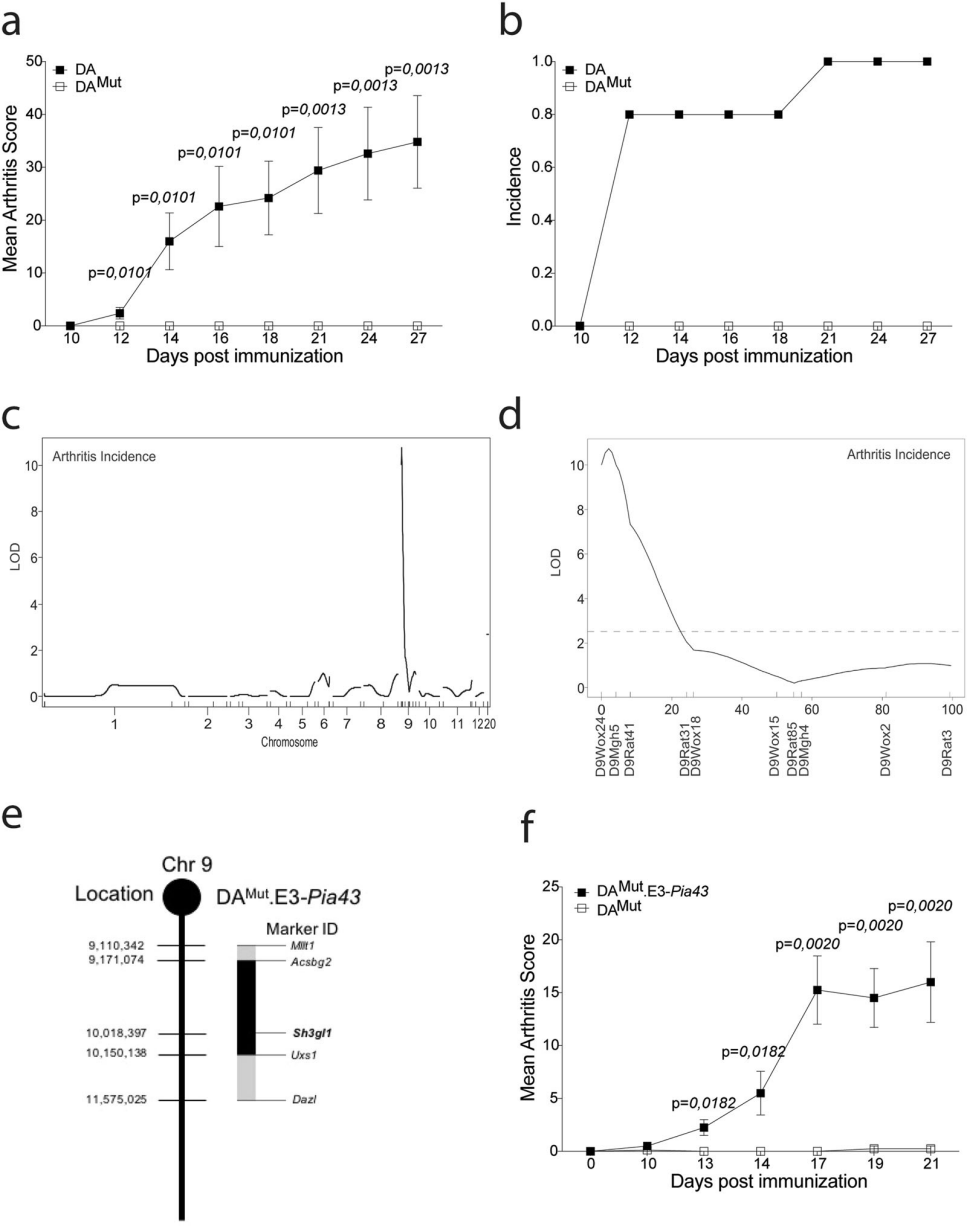
Arthritis regulating loci in the DA^Mut^ rat is restricted to a 2-Mb region on chromosome 9. **a** Mean arthritis score after pristane-induced arthritis in 5 DA and 7 DA^Mut^ rats. The arthritis data has been reproduced three times with the same results. **b** Incidence of arthritis after pristane-induced arthritis in DA and DA^Mut^ rats. **c** LOD score plot for incidence of PIA in 51 (E3.DA-*Pia457*x DA^Mut^) x DA^Mut^ rats of all genotyped chromosomes. **d** LOD score plot for incidence of PIA in (E3.DA-*Pia457* × DA^Mut^) DA^Mut^ rats on chromosome 9, broken line at 2.63 indicate significant linkage **e** Schematic figure of the DA^Mut^.E3-*Pia43* congenic fragment on chromosome 9 with location of the *SH3gl1* gene. Microsatellite markers in the *Acsbg2* and *Uxs1* genes indicate inner border of congenic fragment and markers *Mllt1* and *Dazl* indicate outer borders. **f** Mean arthritis score after pristane-induced arthritis in 8 DA^Mut^ and 4 DA^Mut^ E3-*Pia43* heterozygote littermates. Arthritis has been reproduced in several different congenic fragments with the same results. Non-parametrical Mann–Whitney *U* test was used for statistical evaluation of data. Data are presented as mean with error bars indicating ± SEM with each dot representing an individual value.

The DA rat is commonly used in autoimmunity research due to its high susceptibility to a number of chronic inflammatory disorders such as arthritis and experimental autoimmune encephalomyelitis^11^. We noticed an increased variability in susceptibility to arthritis in our inbred DA rat colony. Arthritis-resistant rats were selected and bred to establish a new line (denoted DA mutated (DA^Mut^)), which was found to be completely protected against pristane-induced arthritis (PIA; Fig. 1a, b). We suspected the underlying cause to be of a genetic origin and not environmental. To test this hypothesis, the DA^Mut^ colony was re-established by cesarean sectioning into a SPF (FELASA II)- controlled facility. We injected the rats with pristane and followed arthritis development. The resulting clinical scores were similar to the conventional facility ruling out obvious environmental factors (Supplementary Fig. 1a, b). To genetically position the mutation, we crossed DA^Mut^ rats to the genetically different, arthritis susceptible, E3.DA-*Pia457* rat strain^12^ and F_2_ offspring from this cross were immunized to induce PIA. Subsequent linkage analysis disclosed a significant association with arthritis incidence for a polymorphic marker on chromosome 9. Typing with additional markers lead to the identification of a quantitative trait locus (*Pia43)*, at the telomeric end of chromosome 9 (Fig. 1c, d). The *Pia43* locus had not been identified in previous E3xDA crosses^13,14^, confirming that this locus was unique to the DA^Mut^ line. The fragment was introgressed into a congenic strain DA^Mut^.E3-*Pia43* and minimized to 2 Mb by new recombinations (Fig. 1e, f).

**Fig. 2:**
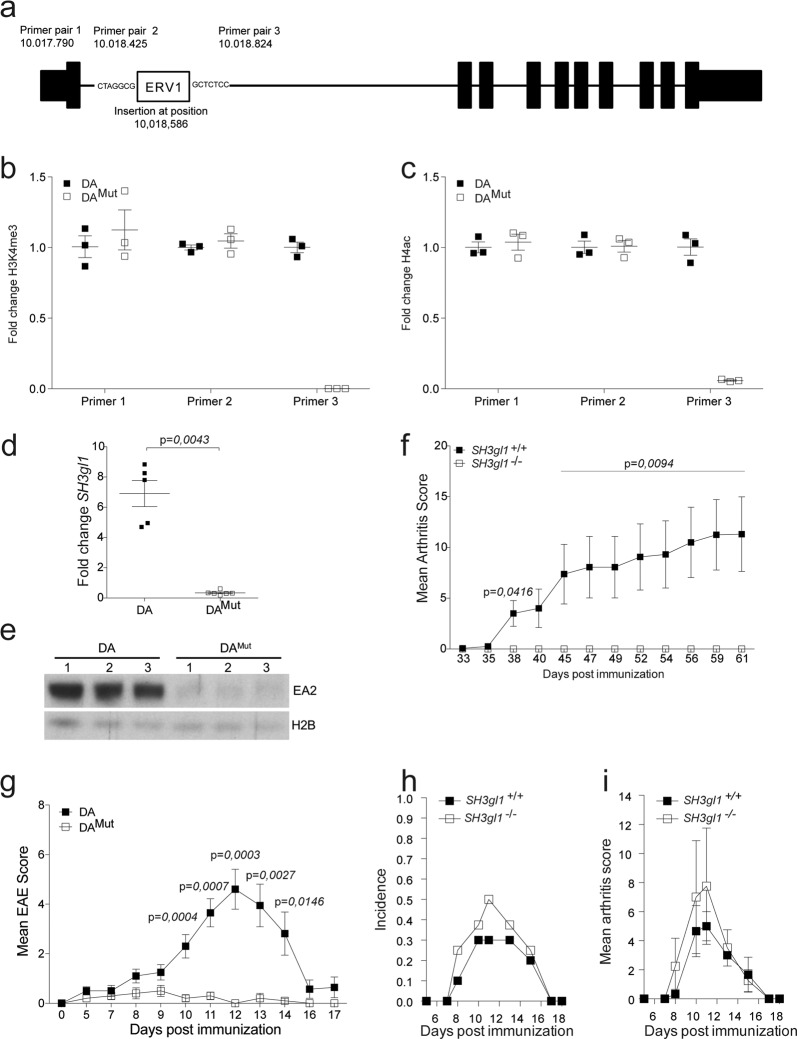
Endophilin A2 deficiency protects against autoimmunity in rodents. **a** Schematic picture of the *SH3gl1* gene with the location of the inserted ERVI long-term repeat element and location of the primer pairs used for quantification of the H3K4me3 and H4Ac levels. **b** Fold change of H3K4me3 levels in 3 DA and 3 DA^Mut^ naïve aged matched rats before (Primer pair 1 and 2) and after (Primer pair 3) the insertion in the *SH3gl1* gene. **c** Fold change of H4Ac levels in 3 DA and 3 DA^Mut^ naïve aged matched rats before (Primer pair 1 and 2) and after (Primer pair 3) the insertion in the *SH3gl1* gene. **d** Fold change of the *SH3gl1* gene expression in PBMCs from 5 DA and 6 DA^Mut^ naïve aged matched rats. Relative fold change calculated to one DA^Mut^ reference sample. **e** Western blot analysis of the expression of the Endophilin A2 protein in brain samples of three DA and DA^Mut^ rats. Histone 2B was used as loading control. **f** Mean arthritis score after collagen-induced arthritis in 9 *SH3gl1* knockouts and 16 wild-type littermates. Arthritis data has been reproduced twice with the same results. **g** Mean EAE score after spinal cord homogenate induced EAE in 10 DA and 10 DA^Mut^ rats. **h** Incidence of collagen-antibody induced arthritis in 10 *SH3gl1*^−/−^ and 10 *SH3gl1*^+/+^ wild-type littermates. **i** Mean arthritis score of sick mice from above experiment after collagen-antibody induced arthritis in 4 *SH3gl1* knockouts and three wild-type littermates. Non-parametrical Mann–Whitney *U* test was used for statistical evaluation of data. Data are presented as mean with error bars indicating ±SEM with each dot representing an individual value.

To identify the specific genetic alteration, we sequenced the DA^Mut^ rat genome and aligned it to the BN rat genome reference (Rno5). All variants in the DA^Mut^.E3-*Pia43* congenic region were manually compared to two previously sequenced DA genomes^15,16^. No single nucleotide polymorphism (SNP) or short insertion nor deletion (indel) was detected that could distinguish DA^Mut^ from the other DA strains in the congenic region, e.g., all 337 SNPs detected between DA^Mut^ and the reference sequence were also found in the other DA genomes. However, a structural variant was revealed, which was unique to the DA^Mut^ rat, and appeared to be the result of an insertion of a long terminal repeat (LTR) element of the ERV class I (ERV1) in intron 1 of the *SH3gl1* gene (Fig. 2a).

Retrotransposons such as LTR elements have been shown to regulate gene expression in both mice and man^17^. To investigate if this was also the case for the ERV1 insertion in the *SH3gl1* gene in DA^Mut^ rats, we determined the levels of lysine 4 methylation in histone 3 (H3K4me3) and the acetylation of histone 4 (H4Ac) using chromatin-immunoprecipitation (ChIP) and qPCR, as these modifications have been associated with active gene transcription^18,19^. We observed that H3K4me3 and H4ac levels upstream of the inserted LTR were similar in DA and DA^Mut^ rats. In contrast, levels in regions situated downstream of the insertion were down-regulated (Fig. 2B, C), indicating that *SH3gl1* gene transcription was not active in DA^Mut^ rats. To determine if this had any impact on gene expression we analysed peripheral blood monocytic cells (PBMCs) from DA and DA^Mut^ rats and quantified gene expression with qPCR. Strikingly, the DA^Mut^ rats had almost no expression of the *SH3gl1* gene compared to DA rats (Fig. 2d). This vastly reduced gene expression also translated to a reduced level of the *SH3gl1* encoded protein EA2 as determined by western blotting (Fig. 2e). To confirm that the arthritis resistance observed in the DA^Mut^ rats was due to a deficiency in EA2 expression we introgressed a deletion of the *SH3gl1* gene into arthritis-susceptible B6N.Q mice through backcrossing and evaluated them with collagen-induced arthritis (CIA). Similar to the DA^Mut^ rats, the *SH3gl1* deficient mice were also protected from arthritis, in contrast to their *SH3gl1* sufficient wild-type littermates (Fig. 2f). To investigate whether the effect was restricted to control only arthritis development or also other autoimmune diseases, we tested experimental autoimmune encephalomyelitis (EAE), a T-cell-dependent model of MS, which confirmed the EA2 mediated protective effect seen in arthritis (Fig. 2g). Additionally, we tested the T-cell independent collagen-antibody induced arthritis model^20^ and observed no difference in disease severity or induction (Fig. 2h, i). We conclude that EA2 is a major regulator of T cell dependent autoimmune disease.

### EA2 deficiency alters the induction threshold of autoreactive T cells

**Fig. 3:**
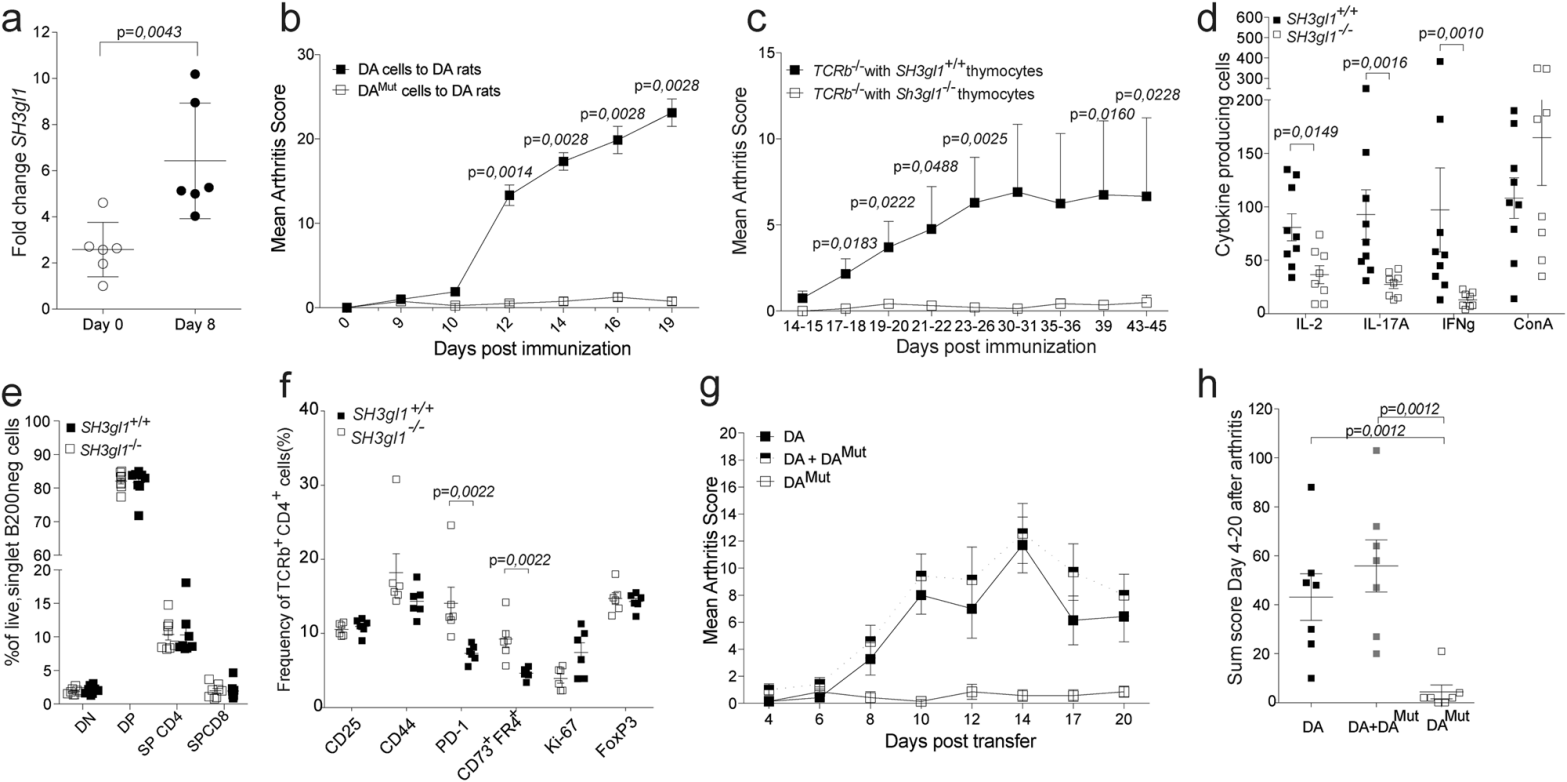
T cells regulate Endophilin A2 mediated protection against arthritis. **a** Expression of *SH3gl1* in sorted T cells from lymph nodes from DA rats at Day 0 and Day 8 after pristane immunization. **b** Mean arthritis score after adoptive transfer of pristane-primed DA and DA^Mut^ cells to 9 respectively 4 naïve DA recipients. The adoptive transfer experiment has been reproduced a total of three times with the same results. **c** Mean arthritis score after GPI-induced arthritis in 17 and 19 TCRβ knockout recipients that had received either *SH3gl1*^−/−^ or *SH3gl1*^+/+^ thymocytes respectively, 7 days prior to disease induction. Data is pooled from three individual experiments. **d** Number of antigen specific IL-2, IL-17A and IFNg producing T cells after recall stimulation with GPI peptide and IL-2 producing cells after ConA stimulation day 44 after GPI-induced arthritis. Data from 9 TCRβ knockout mice reconstituted with wild-type T cells and 8 TCRβ knockout mice reconstituted *SH3gl1* T cells. Recall experiment has been repeated twice in *SH3gl1*^−/−^ mice. **e** Frequency of double negative (DN), double positive (DP), single positive CD4 (SP CD4), and single positive CD8 (SP CD8) thymocytes in 8 *SH3gl1*^−/−^ and 8 *SH3gl1*^+/+^ mice. Data has been reproduced twice. **f** Frequency of CD25, CD44, PD-1, CD73^+^FR4^+^, Ki-67, and FoxP3 positive cells in CD4^+^TCRb^+^ cells from inguinal lymph nodes ten days after GPI-peptide immunization from 6 *SH3gl1*^−/−^ and 6 *SH3gl1*^+/+^ mice. **g** Mean arthritis score in naïve DA recipients after adoptive transfer of pristane-primed DA^Mut^ cells only, DA cells only or DA cells in addition with DA^Mut^ cells, 7 rats per group. **h** Arthritis sum score of the DA recipients above after adoptive transfer of pristane-primed DA cells only or addition with DA^Mut^ cells, 7 rats per group. Non-parametrical Mann–Whitney *U* test was used for statistical evaluation of data. Data are presented as mean with error bars indicating ±SEM with each dot representing an individual value.

Since EA2 deficiency had such a great impact on T cell dependent autoimmune disorders we investigated if the expression of *SH3gl1* increased in T cells after arthritis induction. Indeed 8 days after in vivo activation the expression of *SH3gl1* had more than doubled (Fig. 3a) suggesting an important function of *SH3gl1* during T cell priming and activation. To further elucidate if the protection in *SH3gl1* deficient rats is mediated by T cells we used the CD4^+^ αβ^+^ T cell dependent pristane-induced adoptive transfer model^21^. Pristane-primed lymph node cells from DA or DA^Mut^ rats were expanded for T cells ex vivo before transfer into naïve DA recipients. Only cells from DA rat donors induced severe arthritis (Fig. 3b), indicating that the DA^Mut^ rats were not able to generate arthritogenic T cells. To confirm that the arthritis resistance was intrinsic to T cells, we transferred thymocytes from *SH3gl1* deficient or wild-type littermate mice into TCRβ knockout mice before the induction of glucose-6-phosphate isomerase (GPI) induced arthritis. Mice receiving *SH3gl1* deficient thymocytes developed nearly no arthritis (Fig. 3c) and had fewer antigen-specific cells compared to the wild-type recipients as determined by an in vitro recall assay using the GPI peptide while no significant difference could be seen in mitogen activated cells (Fig. 3d). To determine if this reduction in antigen-specific cells was due to a defect in the thymic T cell development, we assessed thymic T cell populations by flow cytometry. No difference in T cell populations could be observed between the *SH3gl1* deficient mice compared to their wild-type littermates indicating that the reduced numbers of antigen-specific cells were not due to a defect in thymic T cell development (Fig. 3e) but were instead induced in the periphery. To better understand why the *SH3gl1* deficient T cells do not become arthritogenic we looked at activation markers and induction of anergy. While no significant differences in activation markers could be seen ten days after GPI-peptide immunization, the SH3gl1 deficient T cells significantly upregulated anergy markers like PD-1 and CD73/FR4 (Fig. 3f). In a subsequent experiment comparing responses to GPI protein and ovalbumin, the same trend was observed for GPI but not for the exogenous antigen ovalbumin (Supplementary Fig. 2). We next investigated if the *SH3gl1* deficient T cells had a regulatory or suppressive function in the pristane-induced T cell transfer model. Naïve DA rats were given either pristane-primed DA cells alone or together with an equal number of DA^Mut^ cells, with DA^Mut^ cells alone serving as a negative control. No reduction in arthritis severity was evident in recipients receiving pristane- primed DA cells together with DA^Mut^ cells, indicating that DA^Mut^ T cells do not exert their regulatory function via active suppression (Fig. 3g, h) but rather via inhibited activation.

### EA2 deficient rodents develop normally and mount a normal response to cancer and infection

EA2 is a ubiquitous protein with high expression in the central nervous system^22^. Thus, EA2 deficiency could potentially affect many cell types and impact development. However, we did not observe any major physiological disturbances caused by EA2 deficiency in DA^Mut^ rats by general measurements such as blood cell counts, body weight, breeding capacity and life span (up to 1.5 years) in which the DA^Mut^ rats were identical to their normal DA littermates (Supplementary Fig. 3a–e). Similarly to the DA^Mut^ rats, the *SH3gl1* deficient mice develop normally as compared to their wild-type littermates^8^. Given that the EA2 deficient rodents exhibited a profound protection against autoimmune diseases, we assumed that they could have a severely defective immune system. Our animal facility is specific pathogen free but had an outbreak of *S. aureus* caused infections which provoked septic arthritis in immunodeficient mice^23^. However, no *SH3gl1* deficient animals developed a clinical *S. aureus* infection during this outbreak. To further challenge the *SH3gl1* deficient mice towards bacterial infections, an arthritogenic *S. aureus* LS-1 strain^24^ was inoculated intravenously. This septic arthritis model using *S. aureus* LS-1 strain has been shown to be dependent on T cells via activation of T cells by the superantigen, toxic shock syndrome toxin-1 (TSST-1)^25^. In this model of septic arthritis, we did not observe any difference in bacterial clearance or development of arthritis in *SH3gl1* deficient mice compared to wild-type littermates (Supplementary Fig. 3f, g). To further challenge the immune system in EA2 deficient mice, we injected *SH3gl1* deficient and wild-type littermate mice with B16F10 melanoma cells and followed tumor growth. No excessive tumor growth was observed in EA2 deficient mice compared to wild-type littermates (Supplementary Fig. 3h). Thus, EA2 deficiency seems to affect regulation of autoimmunity but does not affect the general health of the rodents nor their susceptibility to a bacterial infection caused by *S. aureus* nor an excess growth of cancer cells.

### EA2 deficiency leads to reduced T cell activation via the TCR

**Fig. 4:**
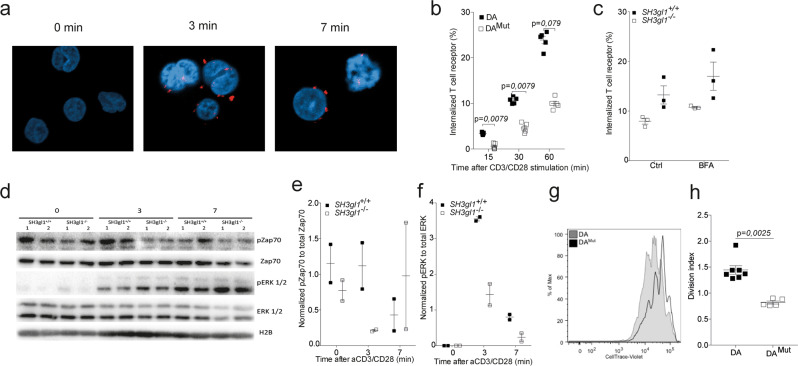
Endophilin A2 co-localizes with the TCR upon activation and regulates TCR internalization and signaling. **a** EA2 co-localization with the TCR in Jurkat cells after anti-CD3/CD28 stimulation determined by proximity-ligase assay and visualized as TexasRed^+^ spots using confocal imaging. **b** Percentage of internalized TCR in T cells from 5 DA and 5 DA^Mut^ rats after anti-CD3/CD28 stimulation. Data has been reproduced two times. **c** Percentage of internalized TCR in CD4^+^CD3^+^ T cells from three *SH3gl1*^−/−^ and *SH3gl1*^+/+^ mice stimulated with anti-CD3/CD28 for 30 min in the presence or absence of Brefeldin A at 1.25 μg/ml. Experiment has been repeated twice with the same results. **d** Representative Western blot analysis from two experiments of phosphorylated and unphosphorylated ZAP70 and ERK1/2 in T cells from two *SH3gl1*^−/−^ and *SH3gl1*^+/+^ mice stimulated with anti-CD3/CD28 for 0, 3, and 7 min. Histone 2B was used as loading control. **e** Normalized OD values of phosphorylated Zap70 compared to unphosphorylated Zap70 from two *SH3gl1*^−/−^ and *SH3gl1*^+/+^ mice. **f** Normalized OD values of phosphorylated ERK1/2 compared to unphosphorylated ERK1/2 from two *SH3gl1*^−/−^ and *SH3gl1*^+/+^ mice. **g** Flow cytometry blot showing in vitro cell proliferation of DA^Mut^ and DA T cells 72 h after anti-CD3/CD28 stimulation. **h** Division index of sorted T cells from 7 DA and 5 DA^Mut^ rats after 72 h of anti-CD3/CD28 stimulation. Non-parametrical Mann–Whitney *U* test was used for statistical evaluation of data. Data are presented as mean with error bars indicating ± SEM with each dot representing an individual value.

Considering the endocytic function of EA2 and the profound effect that loss of EA2 expression had on T cells specifically, we reasoned that EA2 might be important for the internalization of the TCR. Thus, we checked whether EA2 co-localized with the TCR upon activation using a proximity-ligase assay. While no co-localization could be found in un-stimulated cells, we could see that EA2 and the TCR co-localized after only three minutes of anti-CD3/CD28 stimulation (Fig. 4a and Supplementary Fig. 4). We next studied the kinetics of the TCR internalization and indeed, SH3gl1 deficient T cells internalized their TCRs at a slower rate compared to SH3gl1 sufficient T cells (Fig. 4b) with a reduced internalization seen already at 15 min after stimulation. The observed decreased rate of TCR internalization was not due to an increased recycling of TCR as differences in TCR internalization between the SH3gl1 deficient and sufficient T cells sustain even after Brefeldin A treatment (Fig. 4c). To investigate if this had any impact on the responsiveness of the T cells we investigated downstream TCR signaling molecules in SH3gl1 deficient and wild-type T cells. A reduced TCR signaling cascade was observed in SH3gl1 deficient T cells compared to wild-type T cells, with reduced levels of phosphorylated Zap70 and activation of ERK1/2 (Fig. 4d–f). This reduction in responsiveness in the *SH3gl1* deficient T cells translated to reduced T cell proliferation following in vitro stimulation via CD3/CD28 (Fig. 4g, h). The observed reduced proliferation was not the result of reduced internalization of the IL-2 receptor as previously described^26^ but must stem from a reduced activation via the TCR (Supplementary Fig. 5).

### SH3GL1 regulates TCR responses in human T cells and its expression is upregulated in RA patients

**Fig. 5:**
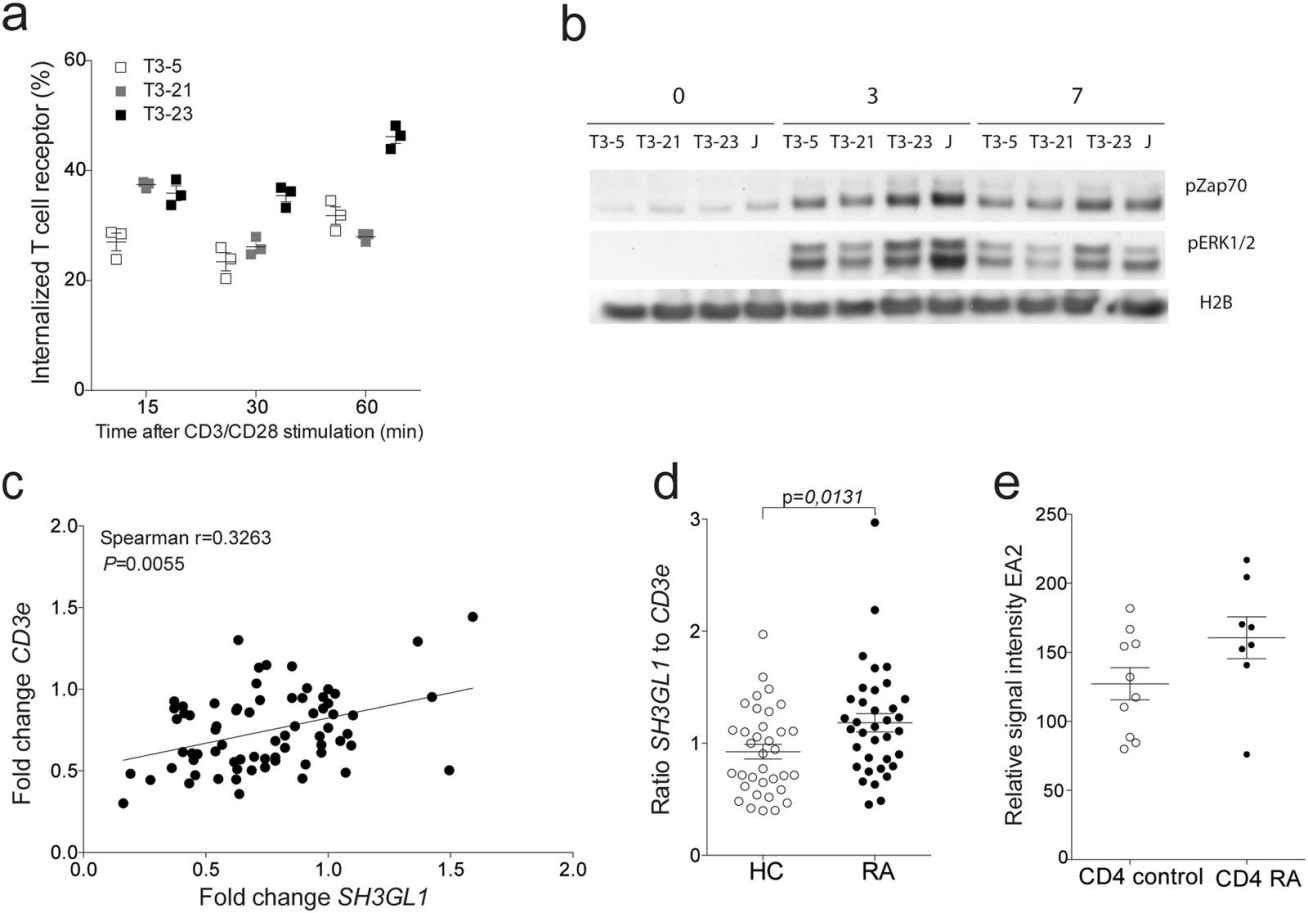
EA2 has a conserved function in human T cells and is upregulated in RA patients. **a** TCR internalization in *SH3GL1* CRISPR knockout Jurkat cells (T3-5 and T3-21) and wild-type control (T3-23). **b** Representative western blot analysis from two experiments of phosphorylated ZAP70 and ERK1/2 in T cells from in *SH3GL1* CRISPR knockout (T3-5 and T3-21), wild-type control (T3-23), and Jurkat (J) cells with anti-CD3/CD28 for 0, 3, and 7 min. Histone 2B was used as loading control. **c** Correlation of *CD3e* and *SH3GL1* gene expression in whole blood of healthy controls and RA patients. Each circle represents one individual. **d** Normalized *SH3GL1* gene expression to *CD3e* gene expression in whole blood of 35 healthy controls and 36 RA patients, randomly selected from the cohort used in **c**. Each circle represents one individual. **e** *SH3GL1* gene expression in sorted CD4^+^ T cells from 10 healthy controls and 8 RA patients (data accessible at NCBI GEO database, accession GSE4588). Non-parametrical Mann–Whitney *U* test was used for statistical evaluation of data. Data are presented as mean with error bars indicating ±SEM with each dot representing an individual value.

Since EA2 is highly conserved and had such a profound effect on T-cell-mediated arthritogenicity in both mice and rats, we next investigated to what extent *SH3GL1* is of importance in human T cells. To demonstrate the relevance of EA2 in human T cells we made *SH3GL1* CRISPR knock-out Jurkat T cells (Supplementary Fig. 6) and stimulated them in vitro with anti-CD3/CD28. Both TCR signaling and TCR internalization were reduced in *SH3GL1* deficient Jurkat cells compared to wild-type control and normal Jurkat T cells (Fig. 5a, b). We addressed the possibility that *SH3GL1* could be overexpressed in T cells from RA patients and determined the expression of both *SH3GL1* and the TCR molecule *CD3e* from whole blood of RA patients and healthy controls and found that the *SH3GL1* expression correlated with expression of the *CD3E* molecule (Fig. 5c). Similar to the findings in animal models, the levels of *SH3GL1* was higher in RA patients when normalized for *CD3E* (Fig. 5d) corroborating the data from the NCBI GEO database^27^ where SH3gl1 gene expression was found to be upregulated in sorted CD4 + T cells from RA patients compared to healthy control (Fig. 5e). Thus, SH3GL1 has a conserved mechanistic function in T cells across species and is relevant in a human disease setting.

## Discussion

Aberrant activation of autoreactive T cells is the key for the self-perpetuating vicious circle of activation of both the innate and adaptive immune response that leads to chronicity and lack of resolution in autoimmune diseases. Using a rat animal model of RA, we discovered SH3GL1 as a major regulator of T cell effector function and autoimmune diseases. We show that deficiency of *SH3gl1* leads to a complete protection against autoimmune diseases in both mutated DA rats and in *SH3gl1* knockout mice and that this results from loss of T cell effector functions. Furthermore, we show that *SH3gl1* expression was increased in T cells during autoimmune arthritis in both rodents and RA patients.

TCR internalization and surface recycling following peptide-MHC recognition on the antigen presenting cell plays a pivotal role in establishing a stable immunological synapse and subsequent T cell activation^28^. To limit potential induction of autoreactive responses, T cell activation is tightly regulated via the TCR and co-stimulatory molecules. Both the surface expression of the TCR as well as the adapters in the TCR signaling cascade are of importance in dictating the effector functions of the T cells. For example, CD4^+^ T cells deficient in the WASH protein show reduced trafficking of the TCR and proliferation of T cells. Similar to *SH3gl1* deficient animals, CD4^+^ conditional WASH knockout mice are also protected from EAE^29^. Several earlier studies demonstrate that SH3GL1 is necessary for internalization and trafficking of a number of receptors^26,30,31^. Our data demonstrate that SH3GL1 also regulates the internalization of the TCR. Because of the importance of TCR internalization and formation of the immunological synapse for efficient TCR signaling, a lack of SH3GL1 leads to reduced T cell signaling and would subsequently result in reduced numbers of autoreactive T cells, in turn limiting the arthritis development. The importance of adapters in the TCR signaling cascade was recently demonstrated by targeting the adapter protein NCK^32^. Like in *SH3gl1* deficient animals, inhibition of NCK reduce proliferation of the T cells and leads to induction of immunosuppression and protection against EAE. Although the regulation of the TCR is different between SH3GL1 and NCK the resulting outcome seems to be similar. Development of effective tissue restricted immunotherapies in the field of autoimmune diseases has proven difficult, with a variety of antigen-specific vaccination strategies developed in the 1990s with limited success^33,34^. Consequently, instead of attempting to address antigen specificity, the field moved more into the depletion of molecules (e.g. TNF blockade^35^) or whole cell populations (e.g. B cells using anti-CD20 antibody^36^) involved in the pathogenesis. Although these therapies have been beneficial they are not curative and there is still a substantial number of patients who are non-responders or develop severe side-effects such as increased susceptibility to infections^37,38^. Our results with *SH3gl1* in rodents demonstrate an interesting pathway by which activation of autoreactive T cells is modulated via the TCR while retaining the ability to respond to pathogens. Thus, SH3GL1 targeting could be a promising therapeutic treatment strategy for RA as well as in other T cell-mediated diseases.

## Methods

### Animals

The E3/ZtmRhd and DA/ZtmRhd rats originated from the Zentralinstitute für Versuchstierzucht, Hannover, Germany and were bred for more than 20 brother/sister mating generations in the animal facility of Medical Inflammation Research lab. The DA^Mut^ rats were isolated from the DA/ZtmRhd stock and bred for more than 10 brother/sister mating generations. To obtain the (E3.DA-*Pia457*x DA^Mut^) DA^Mut^ rats used in the linkage analysis, 2 male DA^Mut^ rats were bred with 4 female E3.DA-*Pia457*^12^ rats. Subsequently, 10 female F_1_ hybrids were bred with 6 DA^Mut^ male rats to produce 51 males of the F_2_ offspring. The C57BL/6.129-Sh3gl1^tm1Pdc^/J^8^, titled SH3gl1^−/−^ in paper, mice were a kind gift from Professor Pietro de Camilli and backcrossed for five generations to the arthritis susceptible C57BL/6 mouse, expressing MHC class II A(q)^39^. C57BL/6.129P2-Tcrb^tm1Mom^/J (Stock No: 002118), titled TCRb^−/−^ in paper, mice were bought from Jackson Laboratory and backcrossed to C57BL/10 expressing the MHC class II A(q) for more than ten generations. All animals included in the experiments were kept in a specific pathogen-free environment following the Federation of European Animal Laboratory Science Association guidelines (FELASA II), in a climate-controlled environment with a 12-h light/dark cycle and fed standard rodent chow and water ad libitum. All animal experiments followed the ARRIVE guidelines, they were performed blindly, age- and sex balanced, mixed in cages and with littermate controls, and approved by the local ethical committees (Malmö/Lund, Göteborg and Stockholm, Sweden, ethical permits M109/07, M107/07, N67/10, N69/10, N169/10, N490/12, N134/13, 353-2012, N35/16, and N288/15).

### Patients and healthy controls

Whole blood from RA patients and healthy controls matched for gender, age and ethnicity were collected in PAX tubes at the Rheumatology Clinic at Karolinska University hospital. RA patients all met the 1987 American college of rheumatology criteria for diagnosing RA. Informed consent was obtained from all the participants and the Stockholm ethical review board approved the study.

### Experimentally induced arthritis

Pristane-induced arthritis (PIA) was induced by a single injection of 100 μl pristane (2, 6, 10, 14-tetramethylpentadecane; ACROS Organics) at the base of the tail. Collagen-induced arthritis (CIA) was induced by injection of 100 μg of pepsin-digested rat collagen type II (as described in^40^) emulsified in 100 μl complete Freund’s adjuvant (Difco) with a boost injection at day 35 with collagen type 2 in incomplete Freund’s adjuvant (Difco). Glucose-6-phosphate isomerase (GPI)-induced arthritis was induced by a single injection of synthetically produced peptide corresponding to the human GPI protein aa325-339(hGPI_325-339_) emulsified in complete Freund’s adjuvant (Difco)^41^. *S. aureus*-induced arthritis was induced by inoculation of the *S. aureus* TSST-1-producing; LS-1 strain intravenously in one of the tail veins with 2.07 × 10^7^ *S. aureus* LS-1/ml in a total volume of 200 μl phosphate-buffered saline (PBS). Arthritis development in PIA, CIA, and GPI experiments were monitored using a macroscopic scoring system. Each limb with the highest possible count of 15, thus with a total possible score of 60 per animal described in more detail for rats in^42^ and for mice in^43^. The *S. aureus*-induced arthritis development was monitored using a macroscopic scoring system where each limb was scored according to a scoring scheme (0, neither swelling nor erythema; 1, mild swelling and/or erythema; 2, moderate swelling and erythema; and 3, marked swelling and erythema). The total score was calculated by adding up all the scores within each animal tested.

### Experimental autoimmune encephalomyelitis

Spinal cord from naïve DA rats was taken and homogenized. EAE was induced by a single injection of spinal cord homogenate emulsified in incomplete Freund’s adjuvant (Difco). EAE was macroscopically scored according to the following scoring scheme 0 = Normal, 1 = Tail weakness, 2 = Tail paralysis, normal gait, 2.5 = Tail paralysis, little affected gait, 3 = Tail paralysis, low back and mild waddle, 3 .5 = Tail paralysis and low back, severe waddle, 4 = Tail paralysis, severe waddle, less sure footing, 4.5 = Tail paralysis, severe waddle, falling and lost balance, 5 = Tail paralysis and paralysis of one limb, crawling, 6 = Tail paralysis and paralysis of a pair of limbs, back is affected, 7 = Tetraparesis, and 8 = Pre-morbid or deceased.

### Adoptive T cell transfer of arthritogenic cells

Eight days after pristane injection, rats were euthanized by CO_2_ inhalation and inguinal lymph nodes were taken and mechanically homogenized through 40μm filters. Cells were washed in PBS and T cells reactivated and expanded in vitro in D-MEM supplemented with HEPES (GIBCO), streptomycin/D-penicillin (104 IU/ml penicillin, 10 mg/ml streptomycin; Invitrogen Life Technologies), β-mercaptoethanol (GIBCO), 5% fetal calf serum (GIBCO) and Concavalin A (3 µg/ml; Sigma-Aldrich). Cells were incubated at 37 °C and 5% CO_2_ for 48 h. Cells were washed in PBS and transferred to naïve DA rats.

### Genotyping and linkage analysis

Toe biopsies were sampled and DNA was prepared by alkaline lysis. The DNA was amplified using fluorescence-marked microsatellites and standard polymerase chain reaction (PCR) reagents (dNTPs, MgCl_2_, Taq Polymerase). The solution was assayed in a PCR Thermal Cycler and the final PCR products were pooled and size-fractioned on a MegaBACE 1000 (Amersham Pharmacia Biotech, Uppsala, Sweden) alternatively on a ABI3730 DNA Analyzer (Applied Biosystems, Life Technologies Corporation, Carlsbad, USA). The data was analyzed using the enclosed program Genetic Profiler 1.1 respectively GeneMapper® Software v4.1 Sequences for microsatellite markers used in the linkage analysis were retrieved from Rat Genome Database (http://rgd.mcw.edu/rgdweb/search/markers.html?100). Additional primers used in mapping the congenic fragments were created using genomic sequences from publicly available rat sequences at NCBI Gene (http://www.ncbi.nlm.nih.gov/gene). Microsattellites were identified and primers were created using the primer design program, Primer3 (http://biotools.umassmed.edu/bioapps/primer3_www.cgi). Primer specificity was controlled by running the sequences through Primer-BLAST at NCBI. (http://www.ncbi.nlm.nih.gov/tools/primer-blast/). Oligos were later ordered from Eurofins MWG Operon. Linkage analysis was performed using R (The R Foundation of Statistical Computing, version 2.0.1) and R/qtl^44^. The normal model and Haley-Knott regression method (1-cM steps) were used for all calculations. Significance threshold of logarithm of the odds (LOD) was determined using permutation tests (*n* = 10000), in which *p* < 0.05 was considered significant. The genetic map was generated in the R/qtl environment based on the recombinations in the cross.

### Chromatin-immunoprecipitation and quantitative PCR

Based on previous ChIP-Seq results from BN and SHR rats^45^, we identified regions with high levels of H3K4me3 modifications in close proximity to the Sh3gl1 gene. Spleen powder (100 mg) from 3 DA and 3 DA^Mut^ rats were used for nuclei isolation and subsequent ChIP analysis was performed according to^45^. The regions of interest were analyzed by ChIP followed by qPCR on ABI 7900HT detection system (Applied Biosystems). PCR primers were designed to amplify designated genomic regions using Primer Express software (Applied Biosystems). qPCR assays were carried out in 384-well plates with a final volume of 20 µL each for 40 cycles. We used Power SYBR Green PCR Master Mix (Applied Biosystems) with diluted ChIPed DNA or un-enriched input DNA as template. Enrichment ratios were calculated according to the 2-∆∆Ct method with endogenous controls (Gapdh) similar to gene expression data sets. For H3K4me3 and H4ac modification see supplementary Table 1 for sequences of primers used for quantification

### RNA extraction and quantitative- polymerase chain reaction

Mononuclear leukocytes from blood (separated from Ficoll-density gradient) or sorted T cells (Pan T Cell MicroBeads, Miltenyi Biotec) were obtained from naïve DA and DA^Mut^ rats and RNA was isolated (Trizol and Pure-link mRNA kit, Invitrogen). Whole blood from RA patients and healthy controls were collected in PAXgene Blood RNA Tubes and total RNA was extracted with the PAXgene Blood RNA kit (PreAnalytiX, Feldbachstrasse, Switzerland) according to the manufacturer’s protocol. cDNA conversion was performed with an iScript cDNA synthesis (Bio-Rad, Hercules, CA, USA). Gene expression experiments were performed using TaqMan gene expression assays(Hs04235263_g1(Sh3gl1), Hs04194521_s1 (PPIA), Hs01062241_m1(CD3e), Rn01527769_g1(Sh3gl1), Rn01527840_m1(Hprt1), Applied Biosystems) and samples were run on a CFX96 RT-PCR(Bio-Rad) according to manufacturers description. Relative fold change was calculated according to the 2-∆∆Ct method after normalization to reference gene, Hprt1 for rat gene expression and PPIA for the human gene expression experiments.

### Western blotting

Whole cell lysates from DA and DA^Mut^ rats were obtained from brain samples by lysis in RIPA buffer (ThermoScientific). Samples were run in a NuPAGE 4–12% Bis-Tris gel (Novex, Invitrogen) and transferred to a PVDF membrane (EMD, Millipore). Blots were blocked with 5% bovine serum albumin (Sigma-Aldrich) in Tris-buffered saline with 0.1% Tween 20 (Cell Signaling Technology) and subsequently incubated with mouse anti-rat Endophilin A2 (clone S51-1, Origene Technologies, Inc) and rabbit anti-histone 2B (Cat. No C49810, LifeSpan Biosciences, Inc). Blot was incubated with peroxidase-conjugated goat anti-mouse IgG(Cat no. 115-035-062, Jackson ImmunoResearch laboratories Inc.) or peroxidase-conjugated donkey anti-rabbit IgG (Cat no.771-036-152, Jackson ImmunoResearch laboratories Inc.) respectively and developed using enhanced chemiluminescence. Pictures including the entire blot can be found in the Source Data file.

### Determining number of antigen-specific cells with enzyme-linked immunospot (ELISpot) assay

Single cell suspension from spleens of mice previously immunized for GPI arthritis was added to plates (MSIPS4W10, Millipore) previously coated with antibodies to IL-2 (clone JES6-IA12), IL-17(clone TC11-18H10) and a-IFNg(AN18) and stimulated for 48 h with 10 μM hGPI_325-339_ in D-MEM supplemented with HEPES (GIBCO), streptomycin/D-penicillin (104 IU/ml penicillin, 10 mg/ml streptomycin; Invitrogen Life Technologies), b-mercaptoethanol (GIBCO), 10% fetal calf serum (GIBCO). Plates were washed and incubated with biotinylated antibodies to IL-2 (clone 5H4), IL-17 (clone TC11-8H4) and IFNγ (R46A2). Plates were washed and Extravidin-Phosphatase (Sigma) was added to wells and incubated for 45 min and washed. Plates were subsequently developed using BCIP/NBT(Sigma). Plates were scanned, analyzed and number of reactive cells were counted with ImmunoSpot software (Cellular Technology Ltd.).

### T cell development

Single cell suspension from thymus of C57BL/6.129-Sh3gl1^tm1Pdc^/J.Q and their wild-type littermates were stained with anti-mouse monoclonal antibodies (B220-Fitc, CD5-PE, CD25-PECy7, TCRb-PerCP5.5, CD4-Q605, CD8-BV650, CD69-APC, CD44-A750) for 20 min at 4 °C. After washing with PBS, cells were acquired on a LSRII (BD Biosciences, Franklin Lakes, NJ, USA) using the BD FACSDiva™ software (BD Biosciences) with gates set to include all viable cells and later analyzed by FlowJo (Tree Star, Inc.) software.

### Cell proliferation assays

T cells from DA and DA^Mut^ lymph nodes were negatively sorted using biotinylated mouse anti-rat CD11b/c (Clone OX42, Biolegend) and mouse anti-rat CD45Ra (Clone OX33, Biolegend) and anti-biotin MicroBeads (Miltenyi Biotec). Sorted T cells were then labeled with CellTrace-Violet (Molecular Probes, Invitrogen) and added to plates coated with anti-rat αβTCR(Clone R73) and anti-rat CD28(Clone JJI319) in D-MEM supplemented with HEPES (GIBCO), streptomycin/D-penicillin (104 IU/ml penicillin, 10 mg/ml streptomycin; Invitrogen Life Technologies), b-mercaptoethanol (GIBCO), 10% fetal calf serum (GIBCO) and incubated at 37 °C, 5% CO^2^. After 72 h cells were washed and later acquired on a LSRII (BD Biosciences, Franklin Lakes, NJ, USA) using the BD FACSDiva™ software (BD Biosciences) with gates set to include all viable cells and later analyzed by FlowJo (Tree Star, Inc.) software.

### TCR internalization assay

Lymph node cells from DA and DA^Mut^ rats were stained with mouse anti-rat CD3 (Clone 1F4) conjugated with Fitc (Biolegend) at 4 °C. Labeled cells were added to plates coated with anti-rat αβTCR (Clone R73) and anti-rat CD28(Clone JJI319) in D-MEM supplemented with HEPES (GIBCO), streptomycin/d-penicillin (104 IU/ml penicillin, 10 mg/ml streptomycin; Invitrogen Life Technologies), b-mercaptoethanol (GIBCO), 10% fetal calf serum (GIBCO), and incubated for 15, 30, and 60 min at 37 °C or left on ice for zero time point. Cells were washed and spilt into two fractions, were one fraction was stripped of surface anti-CD3-Alexa488 antibodies with PBS (GIBCO) pH 2 for 1 min and the other fraction left untreated. For the TCR internalization assay with the CRISPR Jurkat T cells the cells were stained with anti-human CD3 (Clone UTCH1) conjugated with Alexa488 (BD Biosciences) at 4 °C. Labeled cells were added to plates coated with anti-human CD3 (Clone Hit3a, BD Biosciences) and anti-human CD28(Clone CD28.2, BD Biosciences) in RPMI1640 (GIBCO), supplemented streptomycin/D-penicillin (104 IU/ml penicillin, 10 mg/ml streptomycin; Invitrogen Life Technologies) and 10% fetal calf serum (GIBCO) and incubated for 15, 30, and 60 min at 37 °C or left on ice for zero time point. Cells were washed and spilt into two fractions, were one fraction was quenched of surface anti-CD3-Alexa488 signal with unlabeled anti-Alexa488 (MolecularProbes, Invitrogen) and the other fraction left untreated. For the Brefeldin A inhibitor experiment lymph node cells from SH3gl1^−/−^ and SH3gl1^+/+^ mice were stained with Armenian hamster anti-mouse TCRb (Clone H57-597, Biolegend) conjugated with Alexa488 at 4 °C. Labeled cells were added to plates coated with anti-mouse CD3e (Clone 145-2C11, BD Biosciences) and anti-mouse CD28(Clone 37.51, BD Biosciences) in D-MEM supplemented with HEPES (GIBCO), streptomycin/D-penicillin (104 IU/ml penicillin, 10 mg/ml streptomycin; Invitrogen Life Technologies), b-mercaptoethanol (GIBCO), 10% fetal calf serum (GIBCO) and together with 1, 25ug/ml Brefeldin A (Sigma) and incubated for 30 min at 37 °C or left on ice for zero time point. Cells were washed and spilt into two fractions, quenched or no quench, and subsequently stained with rat anti-mouse CD45R-PE-Cy7 (Clone RA3-6B2, BD Biosciences), rat anti-mouse CD4-BV605 (Clone RM4.5, BD Biosciences), rat anti-mouse CD3-PacificBlue (Clone 17A2, Biolegend) and anti-Alexa488 (MolecularProbes, Invitrogen) to half of the samples (quenched). Cells were acquired on a LSRII (BD Biosciences, Franklin Lakes, NJ, USA) using the BD FACSDiva™ software (BD Biosciences) with gates set to exclude doublets and include all viable cells determined as LIVE/DEAD™ -Near-IR (Invitrogen) negative cells and later analyzed by FlowJo (Tree Star, Inc.) software. Percentage of internalized CD3-Alexa488 was calculated as (Qx-Q0)/(Tt-Q0)x100, where Qx is the mean fluorescence of cells quenched with anti-Alexa488 at each time point, Q0 is the mean fluorescence of cells quenched at time zero, and Tt is the mean fluorescence of cells that were not quenched.

### TCR signaling

T cells from lymph nodes and spleens of C57BL/6.129-Sh3gl1^tm1Pdc^/J.Q mice and their wild-type littermates were sorted (Untouched CD4 + T cells MicroBeads, Miltenyi Biotec) and resuspended in D-MEM supplemented with HEPES (GIBCO), streptomycin/D-penicillin (104 IU/ml penicillin, 10 mg/ml streptomycin; Invitrogen Life Technologies), b-mercaptoethanol (GIBCO), 10% fetal calf serum (GIBCO). Cells were rested for one hour at 37 °C and later added to plates coated with 10 μg/ml rat anti-mouse CD3(Clone 145-2C11, BD Biosciences) and 5 μg/ml rat anti-mouse CD28(Clone 37.51, BD Biosciences) and stimulated for 3 and 7 min. Cells were lysed using M-PER(ThermoScientific) supplemented with HALT phosphatase and protease inhibitors (ThermoScientific). Samples were run on a NuPAGE 4-12% Bis-Tris gel (Novex, Invitrogen) and transferred to PVDF membrane (EMD, Millipore). Blots were blocked with 5% bovine serum albumin (Sigma-Aldrich) in Tris-buffered saline with 0.1% Tween (Cell Signaling Technology) and subsequently incubated with rabbit anti- mouse phosphorylated Zap70 (Clone 65E4, Cell Signaling Technology), rabbit anti- phosphorylated-p44/42 MAPK (Erk1/2; Thr202/Tyr204; Cell Signaling Technology) and rabbit anti-histone 2B (Cat no. ab1790 Abcam) as loading control. Blot was incubated with peroxidase-conjugated donkey anti-rabbit IgG (Cat no.771-036-152, Jackson ImmunoResearch laboratories Inc.) and developed using enhanced chemiluminescence. Pictures including the entire blot can be found in the Source Data file.

### Whole genome sequencing and bioinformatics analysis

Genomic DNA from liver from DA^Mut^ rats was isolated using the nuclear lysis buffer, followed by ethanol precipitation of the DNA. DNA integrity was validated with gel electrophoresis. Next generation sequencing was performed at Science for Life laboratory, Stockholm, Sweden using Illumina HiSeq 2000 with 650 bp insert standard DNA (Illumina TruSeq DNA) and paired-end sequencing (2x 100 bp). Sequences were aligned to the BN reference sequence Rno5 and compared to previous sequenced DA genomes^15,16^. Visual inspection of DA^Mut^ sequence alignments in Integrative Genomics Viewer (IGV), Broad Institute, revealed a region of partial or improperly paired alignments in intron 1 of the Sh3gl1 gene and indicated an insertion. The reads aligned in the region and their paired sequences were assembled using Trinity^46^. The inserted sequence was identified as an LTR/ERV1 element, using RepeatMasker (http://www.repeatmasker.org/cgi-bin/WEBRepeatMasker) prediction of the assembled sequences. The insertion was confirmed by Sanger sequencing.

### Colony-forming units in kidneys of S. aureus injected mice

Kidneys were aseptically dissected, kept on ice, homogenized, serially diluted in PBS and spread on blood agar plates. After incubation for 24 h at 37 °C, the number of colony-forming units (CFU) per kidney pair was determined.

### Tumor model

B16F10 melanoma cells were obtained from ATCC and cultured in 5% CO_2_ at 37 °C using 10% deactivated FBS in high glucose DMEM media supplemented with 4.5 grams/ml of glucose, 1 mM pyruvate, 1.5 g of NaHCO3/ 500 ml (Gibo GlutaMAX™, Thermofisher). Cells were washed two times with 37 °C PBS, trypsined with 1X EDTA trypsin, then neutralized with 1:1 ratio of 10% FBS DMEM growth medium to trypsin and 200 µl/mouse of serum free cell growth medium containing 1 × 10^6^ B16F10 (passage 10) p10 melanoma cells were injected subcutaneously. Tumor volume was assessed using an electronic caliper and caliper formula (Length × Width^2^ × 0.52), where the width was the smallest dimension. Maximum allowed tumor size is 1.5cm2 as calculated by (Length × Width^2^ × 0.52).

### Generation of Clustered regularly interspaced short palindromic repeats (CRISPR)/Cas9 – SH3GL1 knock-out Jurkat cells

*SH3GL1* CRISPR knock-out cells were generated and bought from GenScript Ltd. Two clones T3-5 and T3-21 were predicted to be *SH3GL1* knock-outs and T3-23 to be wild type. The three clones were confirmed to be either *SH3GL1* knock-out or wild-type Jurkat cells by and quantitative- polymerase chain reaction and western blotting (Supplementary Fig. 4a, b).

### Proximity- ligase assay

Co-localization of EA2 and TCR was analysed by proximity ligation assay using the Duolink® PLA Starter Kit Mouse/Rabbit from Sigma (Cat. No DUO9210) according to the manufacturer’s description. EA2 was detected using primary rabbit anti-human EA2 (Sigma HPA021485) at 1ug/ml. TCR was detected using primary mouse anti-human CD3 (BD Biosciences Clone Hit3a) at 1ug/ml. Primary antibodies were detected using Duolink® In Situ PLA® Probe Anti-Rabbit PLUS, Affinity purified Donkey anti-Rabbit IgG (H + L) and Duolink® In Situ PLA® Probe Anti-Mouse MINUS, Affinity purified Donkey anti-Mouse IgG (H + L) provided by the kit according to manufacturer’s recommendations. Nuclei were stained using DAPI at 0.5ug/ml for 20 min in PBS. Cells were imaged on a Zeiss LSM 800 confocal laser scanning microscope (Zeiss). TexasRed was detected at λex594nm/λem624nm and DAPI at 358 nm/λem461nm. For image analysis, events were counted using the Analyze Particles function on ImageJ 1.52i. The number of TexasRed positive spots were related to the number of DAPI positive events to give a ratio of TexasRed positive events per cell.

### Statistical analysis

Visualization and calculations of arthritis and EAE data and immunoassays was done using GraphPad Prism v5 software program. The non-parametrical Mann–Whitney *U* test was used for statistical evaluation of arthritis scoring data and immunoassay. P-values < 0.05 were considered significant.

### Reporting summary

Further information on research design is available in the Nature Research Reporting Summary linked to this article.

## Supplementary information

Supplementary Information

Reporting Summary

## Data Availability

All data that support the findings of this study are available in the supplemented data source file and from the corresponding authors. Source data are provided with this paper.

## Source data

Source Data
