# Supplementary material for: Endophilin A2 deficiency protects rodents from autoimmune arthritis by modulating T cell activation
Source: Nat Commun. 2021 Jan 27;12:610. doi: 10.1038/s41467-020-20586-2 (PMC7840939; doi:10.1038/s41467-020-20586-2)
Supplement: Supplementary file 1 — Supplementary Information [file 41467_2020_20586_MOESM1_ESM.pdf]

Supplementary information for

# Endophilin A2 deficiency protects rodents from autoimmune arthritis by modulating T cell activation

**Authors:**

Ulrika Norin*[1], Carola Rintisch[2,1], Liesu Meng[1,3], Florian Forster[1], Diana Ekman[4], Jonatan Tuncel[1], Katrin Klocke[1], Johan Bäcklund[1], Min Yang[1], Michael Y. Bonner[1], Gonzalo Fernandez Lahore[1], Klementy Shchetynsky[5], Maria Bergquist[6], Inger Gjertsson[6], Norbert Hubner[2,7], Liselotte Bäckdahl[1], Rikard Holmdahl*[1,3]

* Corresponding authors

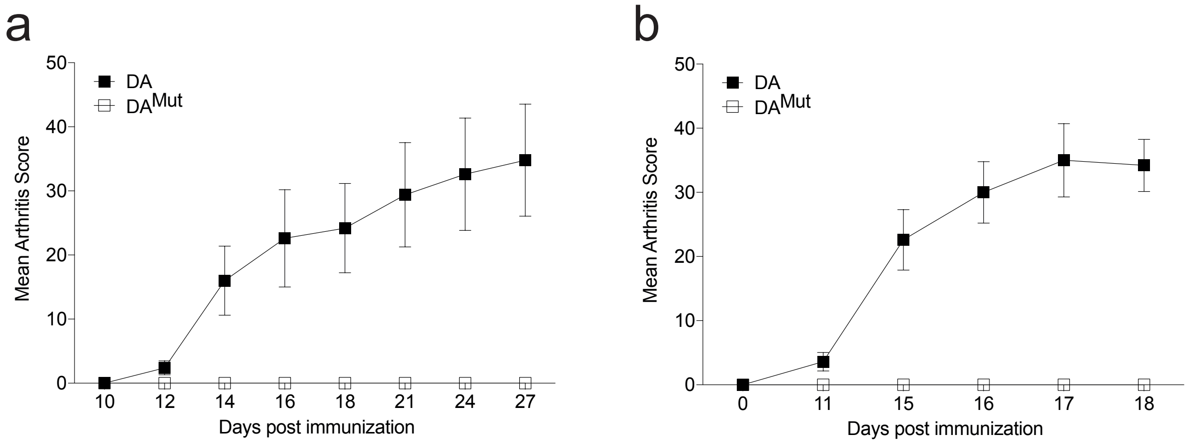


**Supplementary figure 1. Arthritis resistance in DA$^{Mut}$ rat is not due to environmental factors.** a) Mean arthritis score in 5 DA and 7 DA$^{Mut}$ rats after pristane immunization in conventional animal facility. b) Mean arthritis score in 5 DA and 4 DA$^{Mut}$ rats after pristane immunization in SPF (FELASA II) animal facility. Data are presented as mean with error bars indicating ±SEM with each dot representing an individual value.

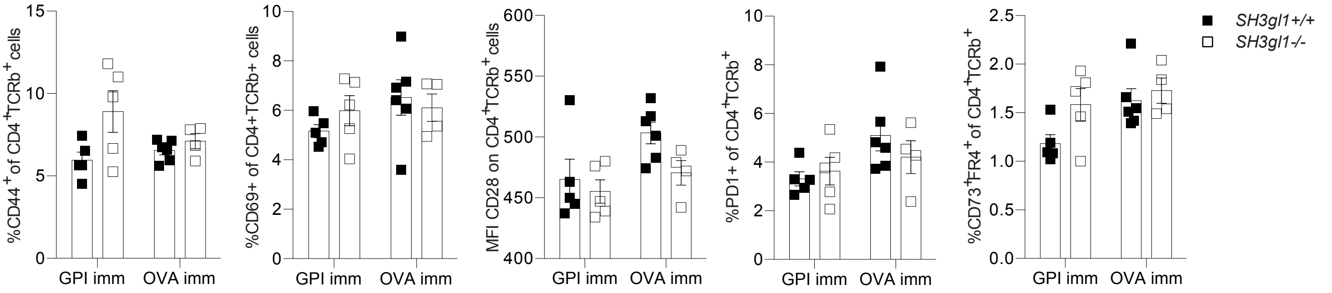


**Supplementary figure 2. No difference in activated and anergic T cells after GPI-protein and ovalbumin immunization.** Frequency and mean fluorescence intensity of activation and anergy markers in CD4+T cells from draining lymph nodes ten days after immunization with GPI protein (5 *SH3gl1* deficient mice and 4 wild type littermates) and ovalbumin (4 *SH3gl1* deficient mice and 6 wild type littermates). Data are presented as mean with error bars indicating ±SEM with each dot representing an individual value.

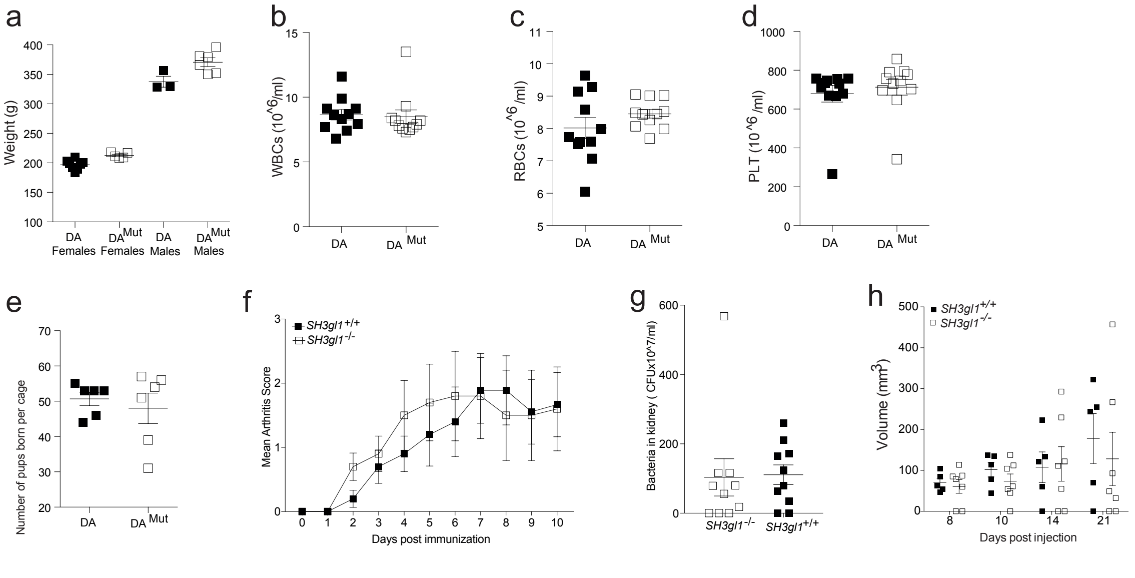


**Supplementary figure 3. EA2 deficiency does not affect the general health of rodents or leads to increased susceptibility to a bacterial infection nor excessive tumor growth.** a) Weight of DA and DA$^{Mut}$ rats at 1.5 years of age b) Number of white blood cells (WBCs) in heparinized blood of 11 DA and 11 DA$^{Mut}$ rats at 1.5 years of age c) Number of red blood cells (RBCs) in heparinized blood of DA and DA$^{Mut}$ rats at 1.5 years of age d) Number of platelets in heparinized blood of DA and DA$^{Mut}$ rats at 1.5 years of age e) Number of pups born from 6 individual DA and DA$^{Mut}$ breeding pairs during a period of approximately one year. f) Mean arthritis score after induction of septic arthritis using *Staphylococcus aureus* LS-1 in 10 *Sh3gl1* deficient and 10 wildtype littermate mice. g) Colony-forming units in kidneys ten days after *Staphylococcus aureus* LS-1 infection. h) Tumor growth in 7 *SH3gl1* deficient mice and 5 wild type littermates after injection of melanoma cells. Data are presented as mean with error bars indicating ±SEM with each dot representing an individual value.

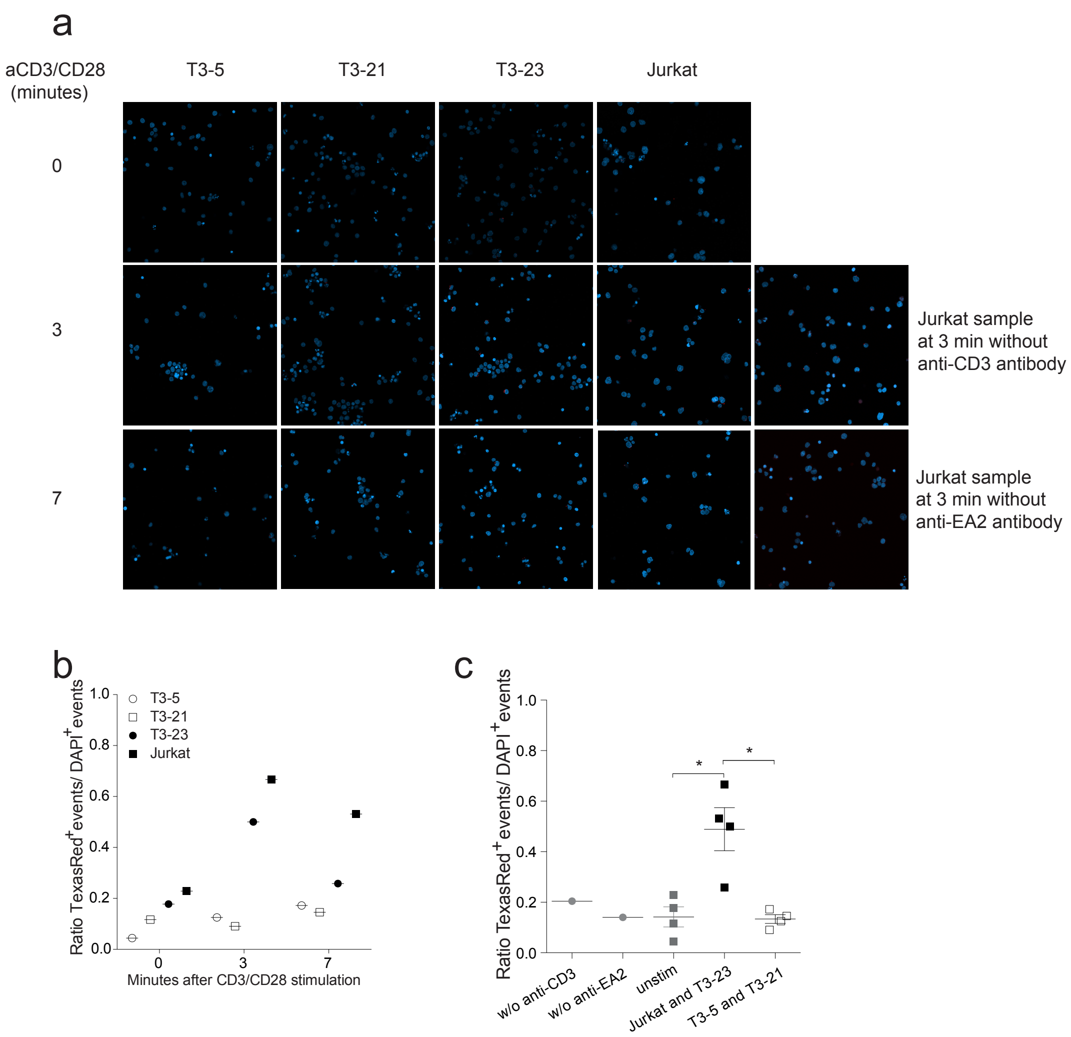


**Supplementary figure 4. Co-localization of EA2 and the TCR in Jurkat and EA2 CRISPR knock-out cells.** a) Detection of EA2 and TCR co-localization in unstimulated and anti-CD3/CD28 stimulated cells with proximity-ligase assay. b) Quantification of number of interactions determined as the ratio of TexasRed positive cells and DAPI positive cells in Jurkat and EA2 knock-out cells. c) Pooled ratio of TexasRed positive cells and DAPI positive cells from the three and seven-minute time points in *SHgl1* sufficient and *SH3gl1* deficient cells compared to the unstimulated samples. Non-parametrical Mann-Whitney *U* test was used for statistical evaluation of data. Data are presented as mean with error bars indicating ±SEM with each dot representing an individual value.

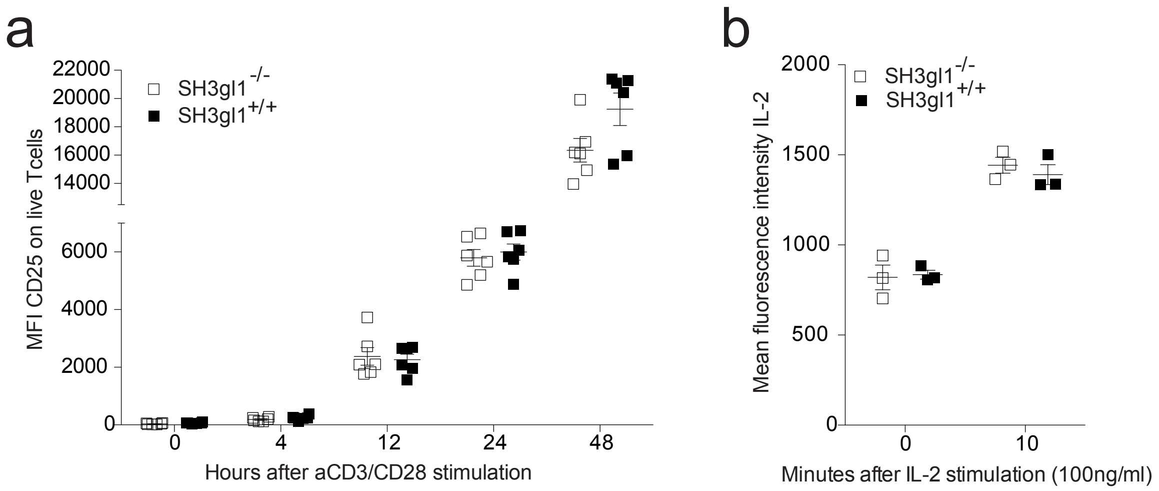


**Supplementary figure 5. EA2 does not regulate activation induced CD25 expression nor IL-2 internalization.** a) Expression of the IL-2 receptor CD25 after anti-CD3/CD28 stimulation in T cells from 6 *SH3gl1*$^{-/-}$ and 6 *SH3gl1*$^{+/+}$ mice. b) Expression of intracellular IL-2 in T cells before and 10 minutes after IL-2 stimulation in 3 *SH3gl1*$^{-/-}$ and 3 *SH3gl1*$^{+/+}$mice. Data are presented as mean with error bars indicating ±SEM with each dot representing an individual value.

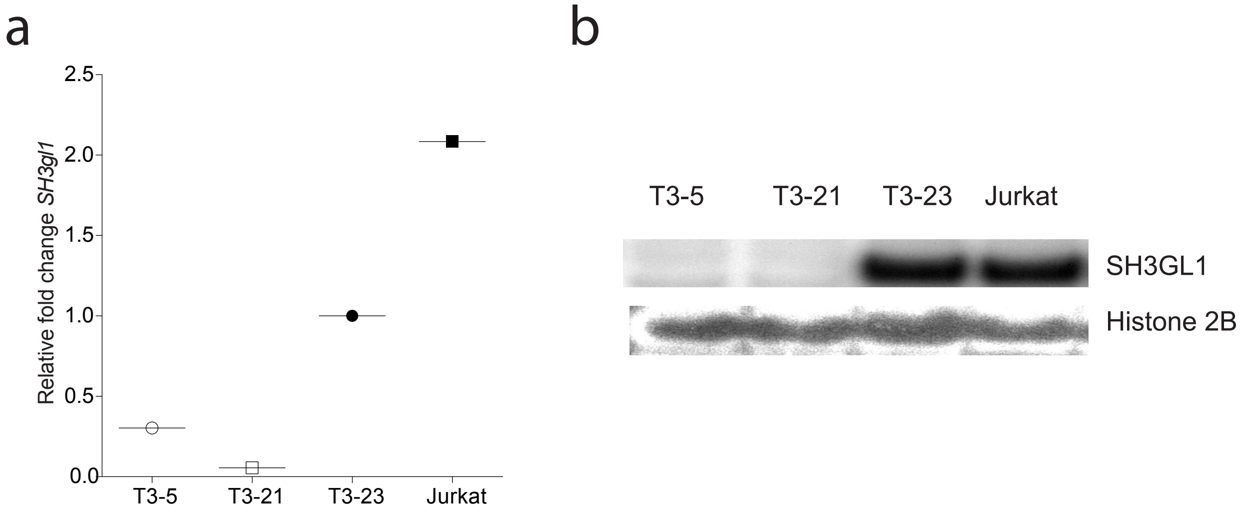


**Supplementary figure 6. Knock-down efficacy of EA2 in EA2 CRISPR knock-out cells.** a) *SH3gl1* gene expression in EA2 CRISPR Jurkat knock-out cells relative to normal Jurkat cells. b) Western blot analysis of the expression of the Endophilin A2 protein in EA2 CRISPR Jurkat knock-out cells. Histone 2B was used as loading control.

a

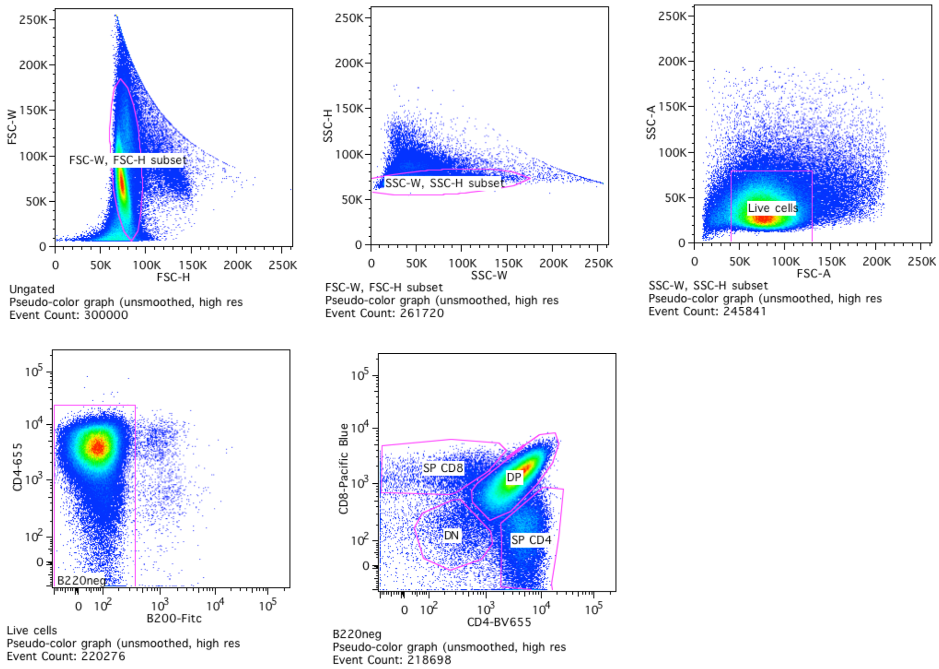
FSC-W
FSC-H
FSC-W, FSC-H subset
Ungated
Pseudo-color graph (unsmoothed, high res
Event Count: 300000
SSC-H
SSC-W
SSC-W, SSC-H subset
FSC-W, FSC-H subset
Pseudo-color graph (unsmoothed, high res
Event Count: 261720
SSC-A
FSC-A
Live cells
SSC-W, SSC-H subset
Pseudo-color graph (unsmoothed, high res
Event Count: 245841
CD4-655
B200-Fitc
B220neg
Live cells
Pseudo-color graph (unsmoothed, high res
Event Count: 220276
CD8-Pacific Blue
CD4-BV655
SP CD8
DP
DN
SP CD4
B220neg
Pseudo-color graph (unsmoothed, high res
Event Count: 218698

b

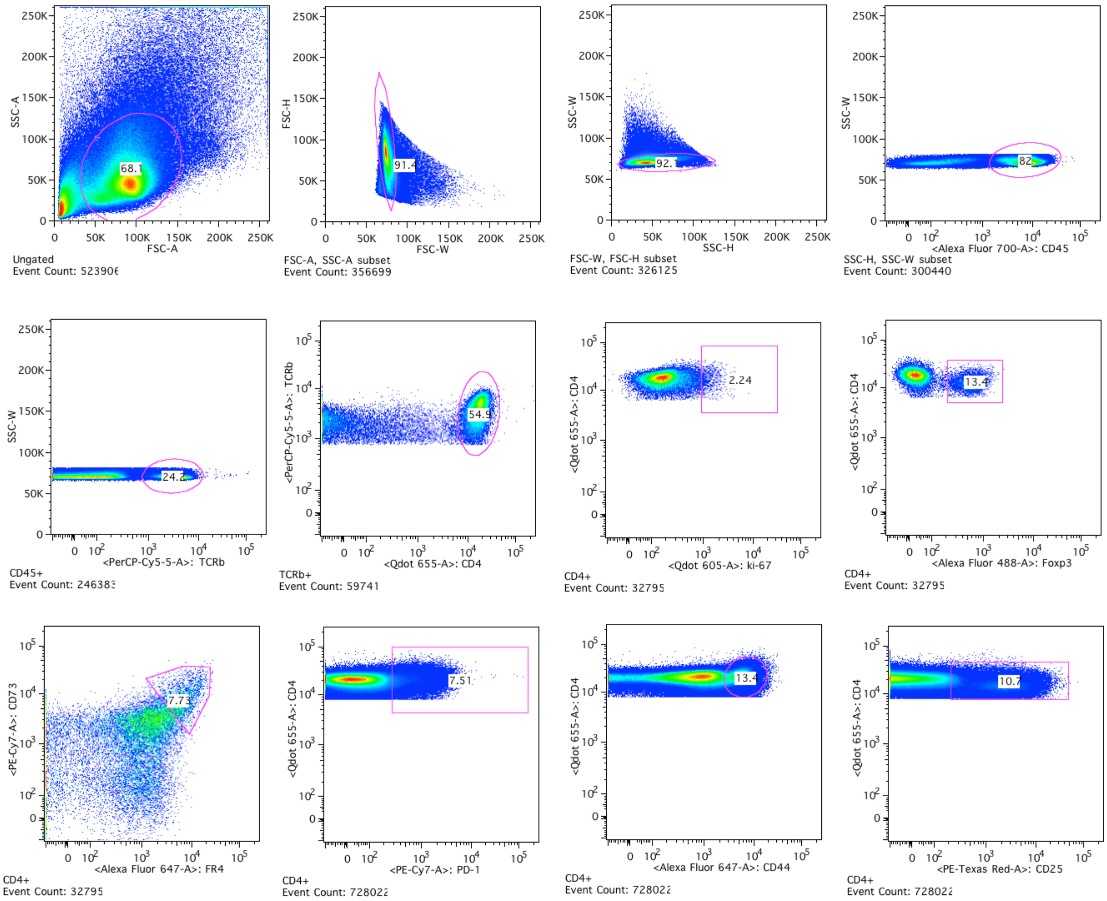
SSC-A
FSC-A
68.1
Ungated
Event Count: 52390
FSC-H
FSC-W
91.4
FSC-A, SSC-A subset
Event Count: 356699
SSC-W
SSC-H
92.1
FSC-W, FSC-H subset
Event Count: 326125
SSC-W
<Alexa Fluor 700-A>: CD45
82
SSC-H, SSC-W subset
Event Count: 300440
SSC-W
<PerCP-Cy5-5-A>: TCRb
24.2
CD45+
Event Count: 24638
<PerCP-Cy5-5-A>: TCRb
<Qdot 655-A>: CD4
54.9
TCRb+
Event Count: 59741
<Qdot 655-A>: CD4
<Qdot 605-A>: ki-67
2.24
CD4+
Event Count: 32795
<Qdot 655-A>: CD4
<Alexa Fluor 488-A>: Foxp3
13.4
CD4+
Event Count: 32795
<PE-Cy7-A>: CD73
<Alexa Fluor 647-A>: FR4
7.73
CD4+
Event Count: 32795
<Qdot 655-A>: CD4
<PE-Cy7-A>: PD-1
7.51
CD4+
Event Count: 728022
<Qdot 655-A>: CD4
<Alexa Fluor 647-A>: CD44
13.4
CD4+
Event Count: 728022
<Qdot 655-A>: CD4
<PE-Texas Red-A>: CD25
10.7
CD4+
Event Count: 728022

c

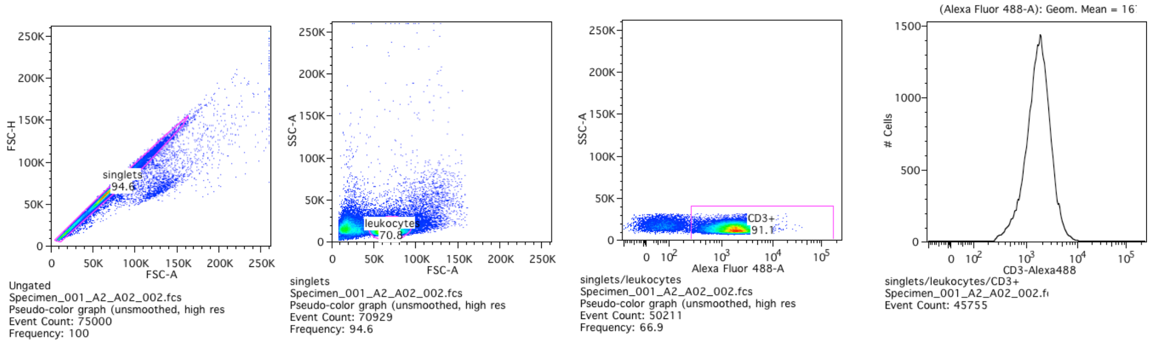
FSC-H
FSC-A
singlets
94.6
Ungated
Specimen_001_A2_A02_002.fcs
Pseudo-color graph (unsmoothed, high res
Event Count: 75000
Frequency: 100
SSC-A
leukocyte
70.8
singlets
Specimen_001_A2_A02_002.fcs
Pseudo-color graph (unsmoothed, high res
Event Count: 70929
Frequency: 94.6
Alexa Fluor 488-A
CD3+
91.1
singlets/leukocytes
Specimen_001_A2_A02_002.fcs
Pseudo-color graph (unsmoothed, high res
Event Count: 50211
Frequency: 66.9
(Alexa Fluor 488-A): Geom. Mean = 16
# Cells
CD3-Alexa488
singlets/leukocytes/CD3+
Specimen_001_A2_A02_002.f
Event Count: 45755

d

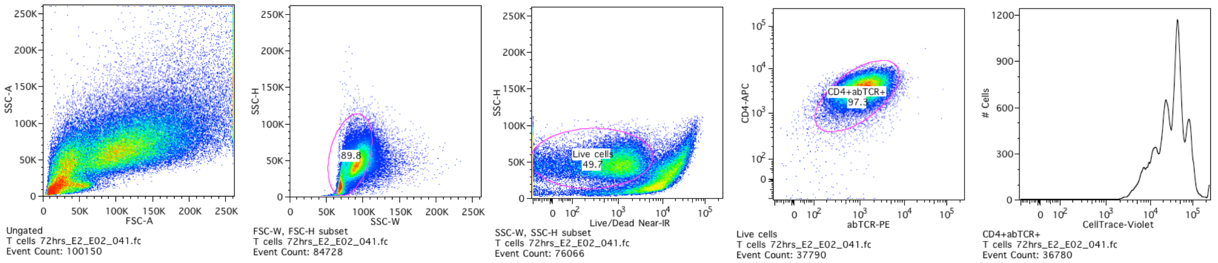
SSC-A
FSC-A
Ungated
T cells 72hrs_E2_E02_041.fc
Event Count: 100150
SSC-H
SSC-W
89.8
FSC-W, FSC-H subset
T cells 72hrs_E2_E02_041.fc
Event Count: 84728
SSC-H
Live/Dead Near-IR
Live cells
49.7
SSC-W, SSC-H subset
T cells 72hrs_E2_E02_041.fc
Event Count: 76066
CD4-APC
abTCR-PE
CD4+abTCR+
97.3
Live cells
T cells 72hrs_E2_E02_041.fc
Event Count: 37790
# Cells
CellTrace-Violet
CD4+abTCR+
T cells 72hrs_E2_E02_041.fc
Event Count: 36780

e

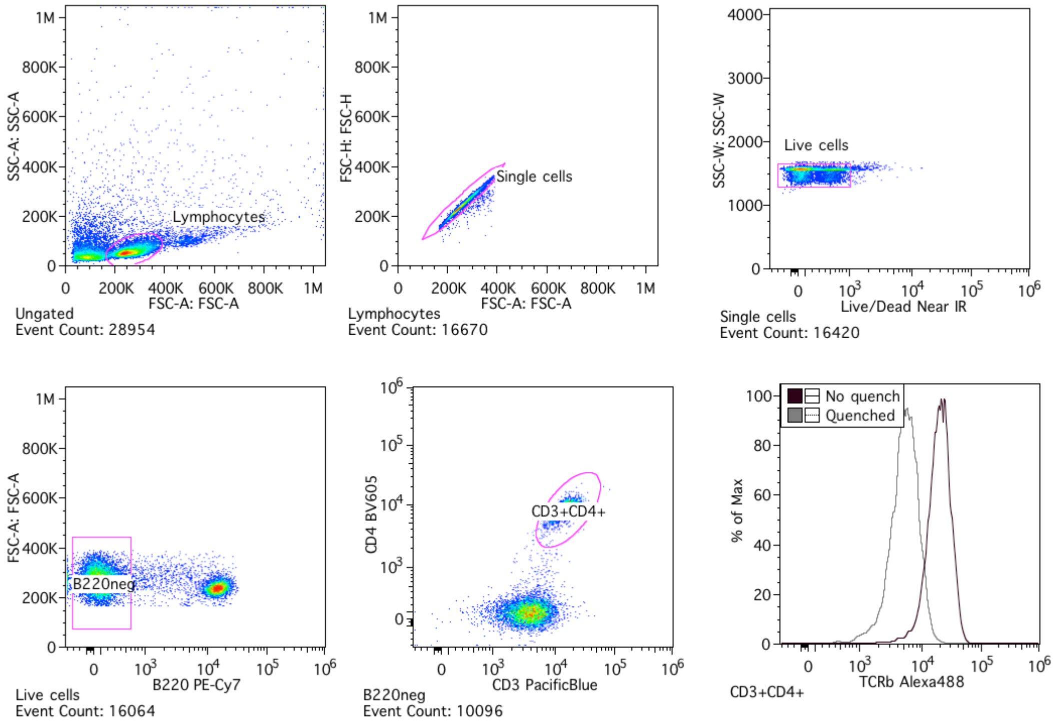
SSC-A: SSC-A
FSC-A: FSC-A
Lymphocytes
Ungated
Event Count: 28954
FSC-H: FSC-H
Single cells
Lymphocytes
Event Count: 16670
SSC-W: SSC-W
Live/Dead Near IR
Live cells
Single cells
Event Count: 16420
FSC-A: FSC-A
B220 PE-Cy7
B220neg
Live cells
Event Count: 16064
CD4 BV605
CD3 PacificBlue
CD3+CD4+
B220neg
Event Count: 10096
% of Max
TCRb Alexa488
No quench
Quenched
CD3+CD4+

f

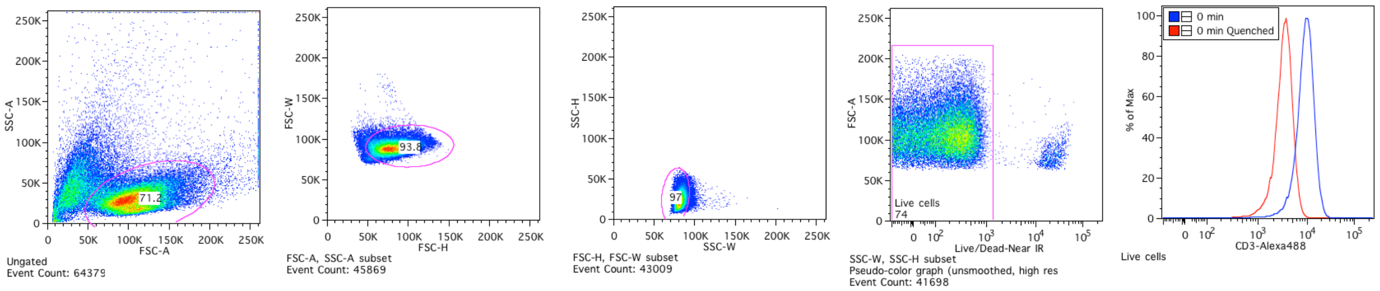

SSC-A
FSC-A
71.2
Ungated
Event Count: 64379
FSC-W
FSC-H
93.8
FSC-A, SSC-A subset
Event Count: 45869
SSC-H
SSC-W
97
FSC-H, FSC-W subset
Event Count: 43009
FSC-A
Live/Dead-Near IR
Live cells
74
SSC-W, SSC-H subset
Pseudo-color graph (unsmoothed, high res
Event Count: 41698
% of Max
0 min
0 min Quenched
CD3-Alexa488
Live cells


g

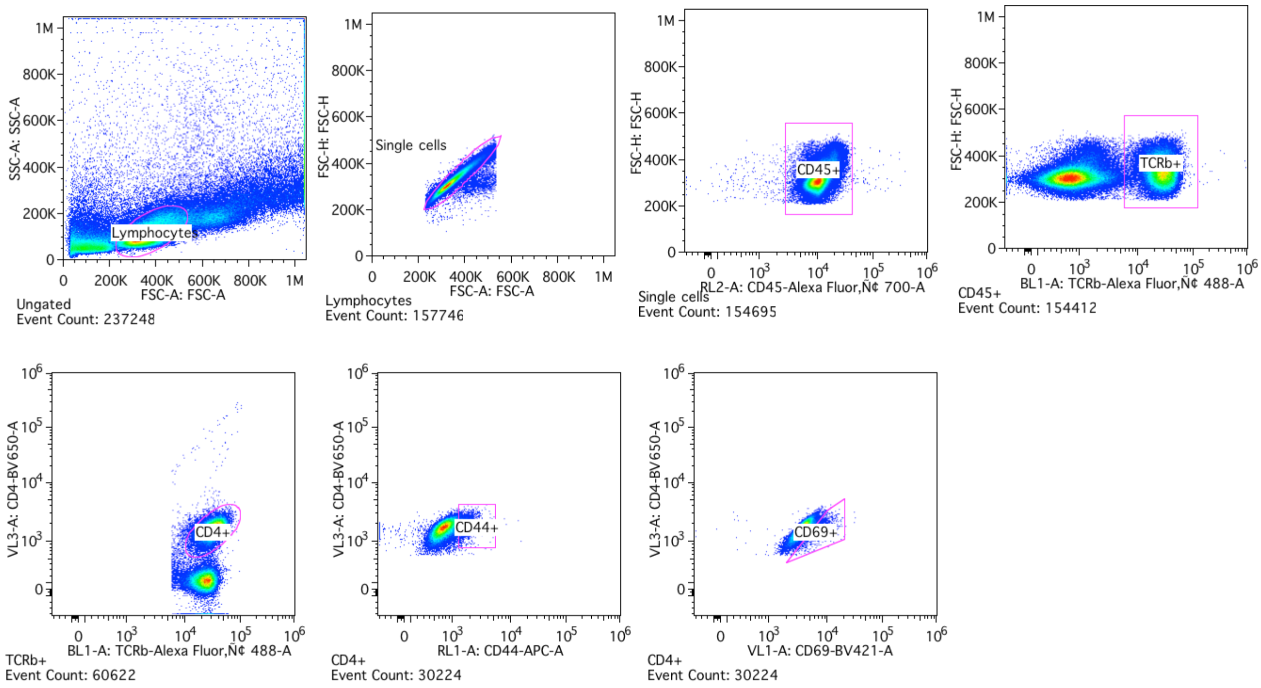

SSC-A: SSC-A
FSC-A: FSC-A
Lymphocytes
Ungated
Event Count: 237248
FSC-H: FSC-H
Single cells
Lymphocytes
Event Count: 157746
RL2-A: CD45-Alexa Fluor,Ñ¢ 700-A
CD45+
Single cells
Event Count: 154695
BL1-A: TCRb-Alexa Fluor,Ñ¢ 488-A
TCRb+
CD45+
Event Count: 154412
VL3-A: CD4-BV650-A
CD4+
TCRb+
Event Count: 60622
RL1-A: CD44-APC-A
CD44+
CD4+
Event Count: 30224
VL1-A: CD69-BV421-A
CD69+
CD4+
Event Count: 30224

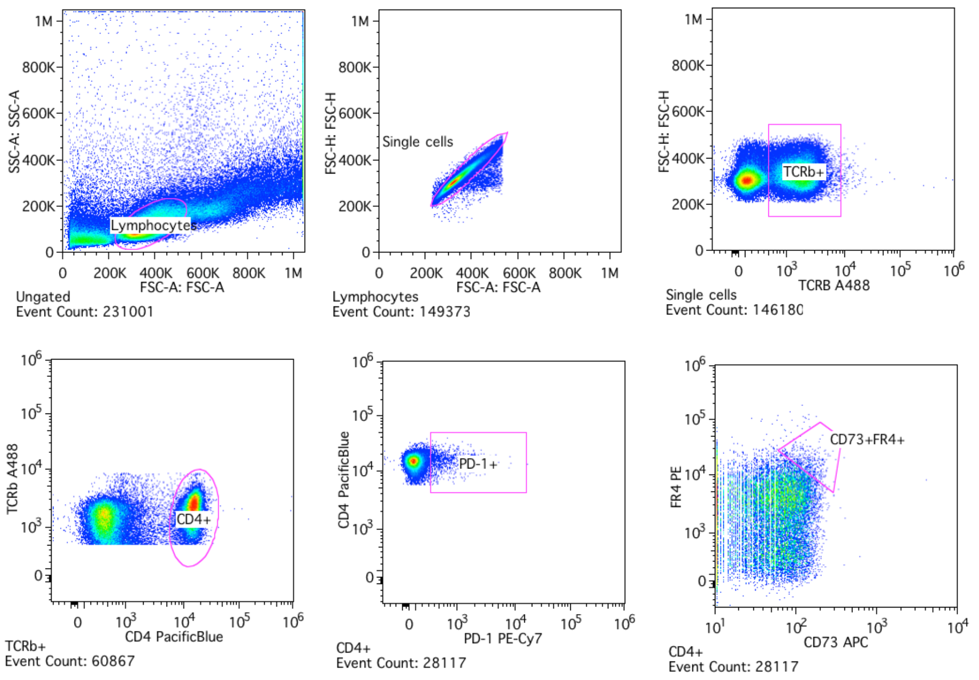
Lymphocytes
Ungated
Event Count: 231001
Single cells
Lymphocytes
Event Count: 149373
TCRb+
Single cells
Event Count: 146180
CD4+
TCRb+
Event Count: 60867
PD-1+
CD4+
Event Count: 28117
CD73+FR4+
CD4+
Event Count: 28117
SSC-A: SSC-A
FSC-A: FSC-A
FSC-H: FSC-H
TCRB A488
TCRb A488
CD4 PacificBlue
PD-1 PE-Cy7
FR4 PE
CD73 APC

h

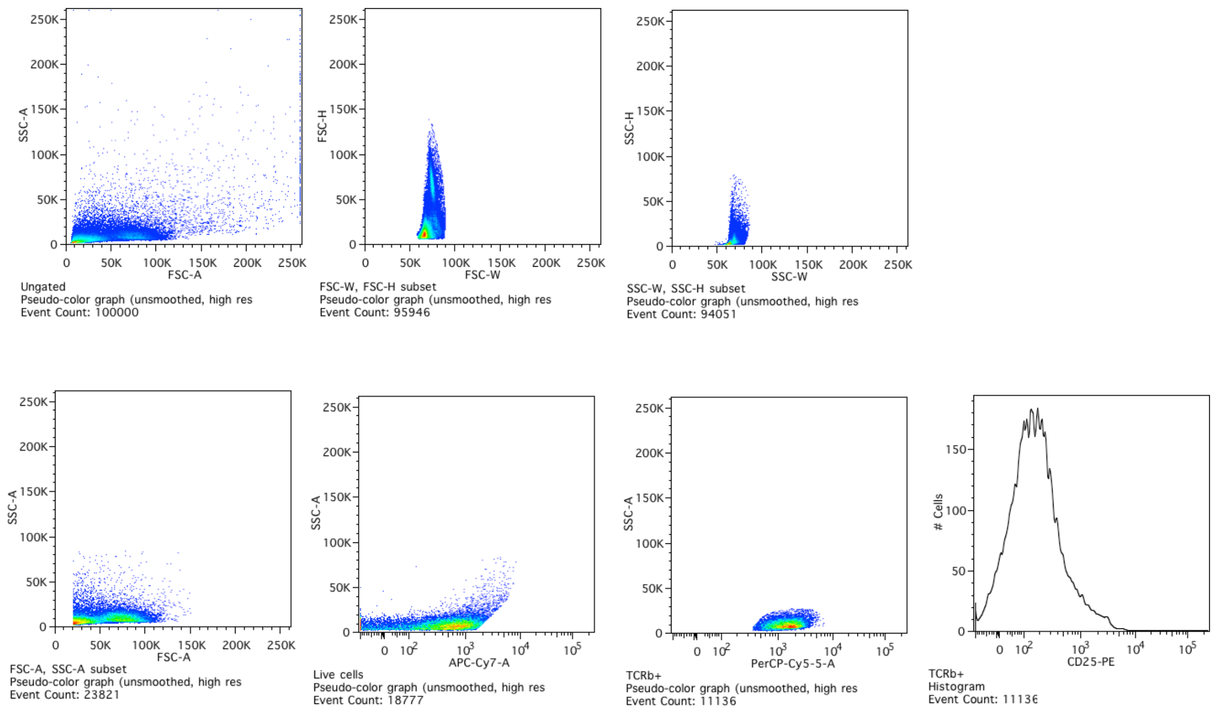
Ungated
Pseudo-color graph (unsmoothed, high res
Event Count: 100000
FSC-W, FSC-H subset
Pseudo-color graph (unsmoothed, high res
Event Count: 95946
SSC-W, SSC-H subset
Pseudo-color graph (unsmoothed, high res
Event Count: 94051
FSC-A, SSC-A subset
Pseudo-color graph (unsmoothed, high res
Event Count: 23821
Live cells
Pseudo-color graph (unsmoothed, high res
Event Count: 18777
TCRb+
Pseudo-color graph (unsmoothed, high res
Event Count: 11136
TCRb+
Histogram
Event Count: 11136
SSC-A
FSC-A
FSC-H
FSC-W
SSC-H
SSC-W
APC-Cy7-A
PerCP-Cy5-5-A
# Cells
CD25-PE

i

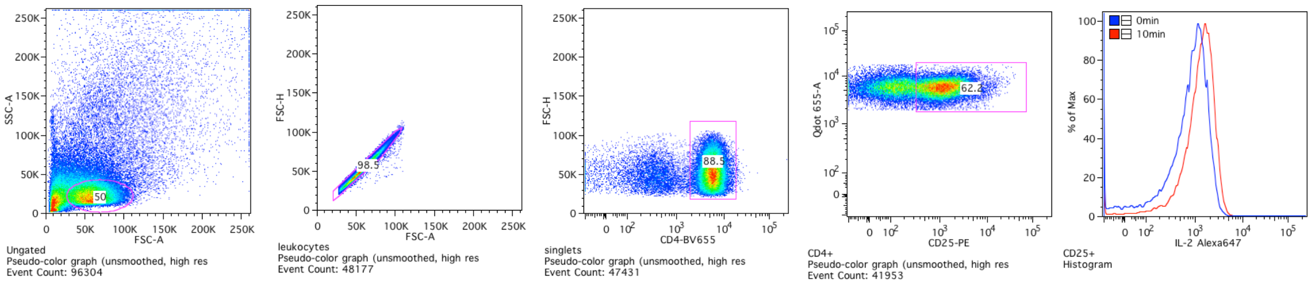


**Supplementary figure 7. Representative FlowJo plots displaying stepwise gating strategy for flow cytometry analysis with subsequent number of cells analyzed within each gate. a)** Gating strategy for thymocyte populations shown in Figure 3e. b) Gating strategy for identification of T cell activation and anergy shown in Figure 3f c) Gating strategy for TCR internalization in rat T cells shown in Figure 4b d) Gating strategy for identification of proliferation of T cells shown in Figure 4f e) Gating strategy for TCR internalization in T cells from lymph nodes shown in Figure 4i f) Gating strategy for TCR internalization in CRISPR Jurkat T cells shown in Figure 5a g) Gating strategy for identification of T cell activation and anergy shown in Supplementary figure 2 h) Gating strategy for identification of expression of CD25 on T cells shown in Supplementary figure 5a i) Gating strategy for identification of intracellular levels of IL-2 shown in Supplementary figure 5b

**Supplementary Table 1. Sequences of PCR primers used for H3K4me3 and H4ac ChIP-qPCR**

| Gene | Primer set | Forward primer | Reverse primer |
|---|---|---|---|
| *Gapdh* | 1 | TACTTCGGCCACCCTATCCA | CATGCCGGTCTGGCTAAATT |
| *Sh3gl1* | 1 | CGAGAAACTGAACTCCGGATCT | GGAACGAATGGTTCTCCAGTTAA |
| *Sh3gl1* | 2 | CGGAAACCTTAGTTCGAGCG | AACTGCTTCTTCAGCCCCG |
| *Sh3gl1* | 3 | CAGGCCACAGAAAATGTTTGTC | GCCCTGTGCAGACTTGGTAAG |
