# Supplementary material for: Endophilin A2 deficiency protects rodents from autoimmune arthritis by modulating T cell activation
Source: Nat Commun. 2021 Jan 27;12:610. doi: 10.1038/s41467-020-20586-2 (PMC7840939; doi:10.1038/s41467-020-20586-2)
Supplement: Supplementary file 2 — Reporting Summary [file 41467_2020_20586_MOESM2_ESM.pdf]

nature research

Corresponding author(s): Ulrika Norin

Last updated by author(s): Nov 5, 2020

Reporting Summary

Nature Research wishes to improve the reproducibility of the work that we publish. This form provides structure for consistency and transparency in reporting. For further information on Nature Research policies, see our Editorial Policies and the Editorial Policy Checklist.

Statistics

For all statistical analyses, confirm that the following items are present in the figure legend, table legend, main text, or Methods section.

n/a	Confirmed	
☐	☒	The exact sample size (n) for each experimental group/condition, given as a discrete number and unit of measurement
☐	☒	A statement on whether measurements were taken from distinct samples or whether the same sample was measured repeatedly
☐	☒	The statistical test(s) used AND whether they are one- or two-sided<br>*Only common tests should be described solely by name; describe more complex techniques in the Methods section.*
☒	☐	A description of all covariates tested
☒	☐	A description of any assumptions or corrections, such as tests of normality and adjustment for multiple comparisons
☐	☒	A full description of the statistical parameters including central tendency (e.g. means) or other basic estimates (e.g. regression coefficient) AND variation (e.g. standard deviation) or associated estimates of uncertainty (e.g. confidence intervals)
☐	☒	For null hypothesis testing, the test statistic (e.g. F, t, r) with confidence intervals, effect sizes, degrees of freedom and P value noted<br>*Give P values as exact values whenever suitable.*
☒	☐	For Bayesian analysis, information on the choice of priors and Markov chain Monte Carlo settings
☒	☐	For hierarchical and complex designs, identification of the appropriate level for tests and full reporting of outcomes
☒	☐	Estimates of effect sizes (e.g. Cohen's d, Pearson's r), indicating how they were calculated

Our web collection on statistics for biologists contains articles on many of the points above.

Software and code

Policy information about availability of computer code

Data collection	*Provide a description of all commercial, open source and custom code used to collect the data in this study, specifying the version used OR state that no software was used.*
Data analysis	*Provide a description of all commercial, open source and custom code used to analyse the data in this study, specifying the version used OR state that no software was used.*

For manuscripts utilizing custom algorithms or software that are central to the research but not yet described in published literature, software must be made available to editors and reviewers. We strongly encourage code deposition in a community repository (e.g. GitHub). See the Nature Research guidelines for submitting code & software for further information.

Data

Policy information about availability of data

All manuscripts must include a data availability statement. This statement should provide the following information, where applicable:

- Accession codes, unique identifiers, or web links for publicly available datasets
- A list of figures that have associated raw data
- A description of any restrictions on data availability

All data that support the findings of this study are available in the supplemented data source file.

Field-specific reporting

Please select the one below that is the best fit for your research. If you are not sure, read the appropriate sections before making your selection.

- [x] Life sciences
- [ ] Behavioural & social sciences
- [ ] Ecological, evolutionary & environmental sciences

For a reference copy of the document with all sections, see nature.com/documents/nr-reporting-summary-flat.pdf

Life sciences study design

All studies must disclose on these points even when the disclosure is negative.

Sample size	Noted in manuscrpt
Data exclusions	A few samples in the human cohort were excluded after being tested as outliers in GraphPad Prism for gene expression, marked in red in the data source file.
Replication	Noted in manuscript
Randomization	Animals of different genotypes were mixed in cages during all animal studies.
Blinding	All animal models studies were scored in a blinded manner, where the person scoring did not know the genotype of the animals.

Reporting for specific materials, systems and methods

We require information from authors about some types of materials, experimental systems and methods used in many studies. Here, indicate whether each material, system or method listed is relevant to your study. If you are not sure if a list item applies to your research, read the appropriate section before selecting a response.

Materials & experimental systems

n/a	Involved in the study	
[ ]	[x]	Antibodies
[ ]	[x]	Eukaryotic cell lines
[x]	[ ]	Palaeontology and archaeology
[ ]	[x]	Animals and other organisms
[ ]	[x]	Human research participants
[x]	[ ]	Clinical data
[x]	[ ]	Dual use research of concern

Methods

n/a	Involved in the study	
[x]	[ ]	ChIP-seq
[ ]	[x]	Flow cytometry
[x]	[ ]	MRI-based neuroimaging

Antibodies

Antibodies used

mouse anti-rat Endophilin A2 (clone S51-1, Origene Technologies, Inc),
rabbit anti-histone 2B (Cat. No C49810, LifeSpan Biosciences, Inc).
goat anti-mouse IgG(Cat no. 115-035-062, Jackson ImmunoResearch laboratories Inc.) peroxidase-conjugated donkey anti-rabbit IgG (Cat no.771-036-152, Jackson ImmunoResearch laboratories Inc.)

mouse anti-rat CD11b/c (Clone OX42, Biolegend)
mouse anti-rat CD45Ra (Clone OX33, Biolegend)
anti-human CD3 (Clone UTCH1) conjugated with Alexa488 (BD Biosciences)
anti-human CD3 (Clone Hit3a, BD Biosciences)
anti-human CD28(Clone CD28.2, BD Biosciences)

hamster anti-mouse TCRb (Clone H57-597, Biolegend) conjugated with Alexa488
anti-mouse CD3e (Clone 145-2C11, BD Biosciences)
anti-mouse CD28(Clone 37.51, BD Biosciences)

rat anti-mouse CD45R-PE-Cy7 (Clone RA3-6B2, BD Biosciences)
rat anti-mouse CD4-BV605 (Clone RM4.5, BD Biosciences)
rat anti-mouse CD3-PacificBlue (Clone 17A2, Biolegend)
anti-Alexa488 (MolecularProbes, Invitrogen)
rabbit anti- mouse phosphorylated Zap70 (Clone 65E4, Cell Signaling Technology)
rabbit anti- phosphorylated-p44/42 MAPK (Erk1/2) (Thr202/Tyr204) (Cell Signaling Technology)
rabbit anti-histone 2B (Cat no. ab1790 Abcam)

peroxidase-conjugated donkey anti-rabbit IgG (Cat no.771-036-152, Jackson ImmunoResearch laboratories Inc.)
rabbit anti-human EA2 (Sigma HPA021485)

Produced in house
anti- mouse IL-2 (clone JES6-IA12),
anti- mouse IL-17(clone TC11-18H10)
anti- mouse IFNg(AN18)
anti- mouse IL-2 (clone 5H4)
anti- mouse IL-17 (clone TC11-8H4)
anti- mouse IFNγ (R46A2)
anti-rat αβTCR(Clone R73)
anti-rat CD28(Clone JJI319)
mouse anti-rat Endophilin A2 (clone S51-1, Origene Technologies, Inc),
rabbit anti-histone 2B (Cat. No C49810, LifeSpan Biosciences, Inc).
goat anti-mouse IgG(Cat no. 115-035-062, Jackson ImmunoResearch laboratories Inc.) peroxidase-conjugated donkey anti-rabbit IgG (Cat no.771-036-152, Jackson ImmunoResearch laboratories Inc.)

mouse anti-rat CD11b/c (Clone OX42, Biolegend)
mouse anti-rat CD45Ra (Clone OX33, Biolegend)
anti-human CD3 (Clone UTCH1) conjugated with Alexa488 (BD Biosciences)
anti-human CD3 (Clone Hit3a, BD Biosciences)
anti-human CD28(Clone CD28.2, BD Biosciences)

hamster anti-mouse TCRb (Clone H57-597, Biolegend) conjugated with Alexa488
anti-mouse CD3e (Clone 145-2C11, BD Biosciences)
anti-mouse CD28(Clone 37.51, BD Biosciences)

rat anti-mouse CD45R-PE-Cy7 (Clone RA3-6B2, BD Biosciences)
rat anti-mouse CD4-BV605 (Clone RM4.5, BD Biosciences)
rat anti-mouse CD3-PacificBlue (Clone 17A2, Biolegend)
anti-Alexa488 (MolecularProbes, Invitrogen)
rabbit anti- mouse phosphorylated Zap70 (Clone 65E4, Cell Signaling Technology)
rabbit anti- phosphorylated-p44/42 MAPK (Erk1/2) (Thr202/Tyr204) (Cell Signaling Technology)
rabbit anti-histone 2B (Cat no. ab1790 Abcam)
peroxidase-conjugated donkey anti-rabbit IgG (Cat no.771-036-152, Jackson ImmunoResearch laboratories Inc.)
rabbit anti-human EA2 (Sigma HPA021485)

Produced in house
anti- mouse IL-2 (clone JES6-IA12),
anti- mouse IL-17(clone TC11-18H10)
anti- mouse IFNg(AN18)
anti- mouse IL-2 (clone 5H4)
anti- mouse IL-17 (clone TC11-8H4)
anti- mouse IFNγ (R46A2)
anti-rat αβTCR(Clone R73)
anti-rat CD28(Clone JJI319)

Validation: All antibodies used in the studies are commercially available and validated

Eukaryotic cell lines

Policy information about cell lines

Cell line source(s)	The CRISPR cell lines were ordered and manufactured by Genscript.
Authentication	The validity of the CRISPR cell lines were checked by qPCR and western blot
Mycoplasma contamination	The cell lines were checked for mycoplasma contamination by the manufactueres but not by us.
Commonly misidentified lines (See ICLAC register)	NA

Animals and other organisms

Policy information about studies involving animals; ARRIVE guidelines recommended for reporting animal research

Laboratory animals: All strains were described in the methods section

Wild animals	The study did not involve wild animals
Field-collected samples	The study did not involve field samples.
Ethics oversight	The animal experiments were approved by a local ethics commitee assigned by the state.

Note that full information on the approval of the study protocol must also be provided in the manuscript.

Human research participants

Policy information about studies involving human research participants

Population characteristics	Not known
Recruitment	Not known
Ethics oversight	The local ethtics commitee.

Note that full information on the approval of the study protocol must also be provided in the manuscript.

Flow Cytometry

Plots

Confirm that:

- [x] The axis labels state the marker and fluorochrome used (e.g. CD4-FITC).
- [x] The axis scales are clearly visible. Include numbers along axes only for bottom left plot of group (a 'group' is an analysis of identical markers).
- [x] All plots are contour plots with outliers or pseudocolor plots.
- [x] A numerical value for number of cells or percentage (with statistics) is provided.

Methodology

Sample preparation	Stated in the methods section
Instrument	LSR II, BD biosciences
Software	FlowJo
Cell population abundance	Shown in the gates of the facs plots.
Gating strategy	Shown in the supporting information 2 file.

- [x] Tick this box to confirm that a figure exemplifying the gating strategy is provided in the Supplementary Information.
